# Continuous glucose monitor use with and without remote monitoring in pregnant women with type 1 diabetes: A pilot study

**DOI:** 10.1371/journal.pone.0230476

**Published:** 2020-04-16

**Authors:** Sarit Polsky, Rachel Garcetti, Laura Pyle, Prakriti Joshee, Jamie K. Demmitt, Janet K. Snell-Bergeon

**Affiliations:** 1 Barbara Davis Center, University of Colorado Anschutz Medical Campus, Aurora, Colorado, United States of America; 2 Department of Pediatrics, School of Medicine, University of Colorado Anschutz Medical Campus, Aurora, Colorado, United States of America; University of Auckland, NEW ZEALAND

## Abstract

**Background:**

To examine whether continuous glucose monitoring (CGM) with remote monitoring by followers (family/friends) changes glucose management, follower interventions, and health outcomes compared to CGM alone in pregnant women with diabetes.

**Methods:**

We prospectively stratified first trimester pregnant women with Type 1 Diabetes to CGM Share (remote monitoring) or CGM Alone. We enrolled a main follower per woman. We retrospectively acquired data for pregnant women who did not use CGM (no CGM). We compared hemoglobin A1c (HbA1c) between groups. We compared sensor glucose, follower interventions, and gestational outcomes between CGM Alone and CGM Share. Longitudinal mixed effects models were used for analyses of changes in outcomes over time.

**Results:**

HbA1c decreased in all groups throughout pregnancy and was significantly lower over time in women using CGM Share (n = 15) compared to CGM Alone (n = 13) or no CGM (n = 8) (p = 0.0042). CGM Share users had lower median sensor glucose levels (p = 0.0331) and percent time spent >180 mg/dL (p = 0.0228) across pregnancy. There were no significant differences in maternal and fetal outcomes between groups. CGM Share followers had more alerts for hypoglycemia, but did fewer interventions.

**Conclusions:**

In this small pilot study, use of CGM with remote monitoring improved some glycemic metrics in pregnant women with diabetes.

## Introduction

Pregnancies complicated by Type 1 Diabetes (T1D) are associated with high risk for adverse maternal and fetal outcomes [1, 2]. It is recommended that women maintain intensive glucose management with frequent insulin dose adjustments to achieve target glucose and hemoglobin A1c levels (HbA1c) throughout gestation while also avoiding severe hypoglycemia [1, 3]. One tool that can help in the management of T1D during gestation is the continuous glucose monitor (CGM).

Early studies of CGM use in pregnancy were inconclusive regarding potential reductions of adverse gestational outcomes [4–8]. In the CONCEPTT study, a large randomized controlled trial of gestational use of CGM in women with T1D, CGM use had benefits over self-monitoring of blood glucose alone with respect to neonatal health (lower incidence of large-for-gestational age [LGA] infants, neonatal hypoglycemia, and neonatal intensive care unit admissions) [9]. Yet, in this group with relatively low HbA1c levels (HbA1c 6.35% CGM group vs 6.53% Control group at 34 weeks gestation, p = 0.0372), these benefits seem to be derived from an increase in the time in the target glucose range and a decrease in post-prandial hyperglycemia [10], highlighting that HbA1c alone may not be the best measure of optimal gestational glucose control. It is likely that the CGM data guided insulin therapy, particularly doses applicable to prandial insulin delivery, which lead to the higher time spent in the pregnancy target glucose range. Moreover, the CGM’s ability to help individuals identify glucose trends and receive alerts for hyperglycemia and hypoglycemia before doing a scheduled point-of-care glucose check decreases glucose variability [11, 12], which may ultimately affect outcomes.

Remote monitoring of glucose data has the potential to improve health outcomes during pregnancy. Remote monitoring refers to the ability of someone other than the patient to view data from glucose meters, CGMs, and/or insulin pumps [8]. Previous studies in pregnancy have focused on providers having remote access to data and then adjusting treatment in women with gestational diabetes mellitus or T1D [13–16]. We examined whether the use of remote glucose monitoring by followers (family/friends) would improve maternal glucose management and gestational health outcomes compared to use of CGM alone in pregnant women with T1D.

Our primary objective was to assess the role of CGM usage either alone or with remote monitoring capabilities (Share^™^) among women with T1D associated with pregnancy. Secondary objectives were to: 1) assess remote monitoring capabilities of followers (Share^™^ usage) that lead to interventions for hypoglycemia and hyperglycemia that affect subjects wearing CGM, 2) measure changes in HbA1c from baseline to 3 months with maintained control at 6 and 9 months, and 3) assess effects of CGM usage on maternal and fetal health outcomes (eclampsia/pre-eclampsia, live birth rates, birth weight, neonatal hypoglycemia, and other similar measures).

## Materials and methods

### Study design and stratification

This single-center, open-label, non-randomized, investigator-initiated pilot study recruited three cohorts of participants: (1) women with T1D were prospectively enrolled within the first trimester of gestation or while planning pregnancy (preconception), (2) followers (family or friends) of pregnant women with T1D in the first cohort were prospectively enrolled, and (3) women with T1D who had a pregnancy within the past 3 years of the date enrollment began for the first cohort, who did not use CGM therapy during gestation, and who had available retrospective data. All participants had to be ≥18 years of age. Additional inclusion criteria for the pregnant women and the women planning pregnancy included gestational age ≤13 weeks or intent to conceive within the next 6 months, T1D duration >1 year, willingness to do 3–7 blood glucose measurements daily, Multiple Daily Injection (MDI) therapy or Continuous Subcutaneous Insulin Infusion (CSII) therapy, and ability to speak, read, and write English. Exclusion criteria included extensive skin changes/diseases that inhibit wearing a sensor on normal skin and known severe allergy to adhesives within the last 3 months. For women in the retrospective cohort (no CGM arm), additional inclusion criteria were no CGM use during gestation or <2 months of use after the first visit, ≥1 clinic visit each trimester, and ≥6 pregnancy visits throughout gestation. This protocol was approved by the Western Institutional Review Board on August 13, 2015 and registered on clinicaltrials.gov (NCT02556554). There was a delay in registering this study due to staffing issues and account set-up in the registry system. The authors confirm that all ongoing and related trials for this intervention are registered. All participants provided written informed consent.

Women in the first trimester of pregnancy were assigned to one of three groups: (1) CGM use with Share (CGM Share): women with iPhone. iPad, or iPod Touch and followers with devices compatible for data viewing, (2) CGM use alone (CGM Alone): women without iPhone, iPad, or iPod Touch, and (3) no CGM use: women who did not want to use a CGM in pregnancy. Assignment was based on two factors: device compatibility and willingness to wear a CGM. Women willing to wear a CGM who had a device compatible with CGM Share were enrolled in the CGM Share arm, while those with incompatible devices were enrolled into CGM Alone. Women unwilling to wear a CGM could continue in the prospective study in the no CGM arm. In the event that there would be few women enrolling in the no CGM arm, the retrospective cohort data would be used. Women seen at the Pregnancy and Women’s Health Clinic of the Barbara Davis Center for Diabetes for preconception counseling or a first pregnancy visit were recruited between August 27, 2015 and June 16, 2016.

### Study procedures

Pregnant participants in the prospective cohorts were trained on the use of the Dexcom G4 Platinum® CGM system with Share^™^ (CGM Share) or without Share^™^ (CGM Alone) at the first pregnancy visit. Share^™^ is a Bluetooth® low energy secure wireless communication system that allows remote viewing of sensor glucose levels, trends, and data between the person with diabetes wearing the CGM and her designated family members/friends. The followers can receive alerts (alarms) for pre-specified low and high sensor glucose values. The person with diabetes can designate herself as a follower to allow data viewing through approved devices. Women were given the Dexcom G4 system and sensors throughout pregnancy. Women who already had the system or who were using Dexcom G5, were given sensors throughout pregnancy.

Pregnant women were seen at the Barbara Davis Center Pregnancy and Women’s Health Clinic at least once each month during gestation, and 4–6 weeks post-partum, for routine peri-partum care and study visits. A visit up to 3 months post-partum was acceptable for a final study visit. A questionnaire was administered at baseline to obtain data about demographics and participant medical history. A point-of-care HbA1c was checked at each study visit. Glucose meters, insulin pumps, and CGM data were downloaded at each study visit. Women were asked to document each use of acetaminophen and related products as an event marker in the CGM and on paper. For patients on MDI, study investigators recorded basal and bolus insulin doses at each study visit. A 7-point profile, self-monitored blood glucose values checked before and after meals and at bedtime, was obtained once each trimester. Additional questionnaires included the hypoglycemia fear survey [17] (at baseline in the 1^st^ trimester, once in the 2^nd^ trimester, once in the 3^rd^ trimester, and post-partum) and a post-partum questionnaire inquiring about the labor and delivery process, maternal and fetal complications before and after delivery, baby’s anthropometrics at birth (weight and length), baby’s current age, breastfeeding, and other measures. Medical records from the labor and delivery admission were reviewed, when available.

Severe hypoglycemia was defined as hypoglycemia (glucose <70 mg/dL) requiring the assistance of a third party. Birth weight centile was calculated using the Customised Centile Calculator from Gestation Network using GROW software [18]. Small-for-gestational age was defined as birth weight <10^th^ percentile and LGA as birthweight >90^th^ percentile, individually adjusted for maternal characteristics (race/ethnicity, height, weight, parity) and infant characteristics (gestation-adjusted birth weight and sex) [18]. Macrosomia was defined as infant birth weight ≥8.8 pounds (4 kilograms). Neonatal hypoxia was defined as the neonatal need for oxygen after birth as per participant report in the post-partum questionnaire or review of hospital records.

Followers of prospectively enrolled pregnant participants were given instructions for downloading the follower app on their smart phones and for setting alerts. They were administered a questionnaire monthly throughout gestation inquiring about the frequency of viewing information about glucose trends, receiving alerts or information about extreme low and high glucose values, and acting upon aforementioned data to assist the participant. Participant study procedures took place between August 27, 2015 and May 1, 2017.

### Statistical analysis

Participants who had a miscarriage and did not become pregnant again, who dropped out of the study, or who were withdrawn were excluded from analyses. CGM data within 12 hours after use of acetaminophen use were omitted. The CGM time in range in pregnancy of 63–140 mg/dL was used per the International Consensus Report [19]. Groups were compared using t-tests, ANOVA, or Kruskal-Wallis tests for continuous variables, and Fisher exact tests for categorical variables. Longitudinal mixed effects models were used for analyses of the change in maternal CGM measures over time. The time variable (month of pregnancy) was treated as a categorical variable to allow for nonlinear trajectories over time. For each outcome, a model with an interaction between group and time was fit to test whether the trajectory of the outcome differed between the groups. If the interaction term was not significant, it was removed. The models were repeated while adjusting for the preconception value of the outcome, if available. Maternal and fetal outcomes in the groups were compared using linear and logistic regression. The models for maternal and fetal outcomes were adjusted for maternal smoking at baseline, which was significantly different between the groups. Study data were collected and managed using REDCap electronic data capture tools hosted at the University of Colorado Denver [20].

## Results

**Fig 1:**
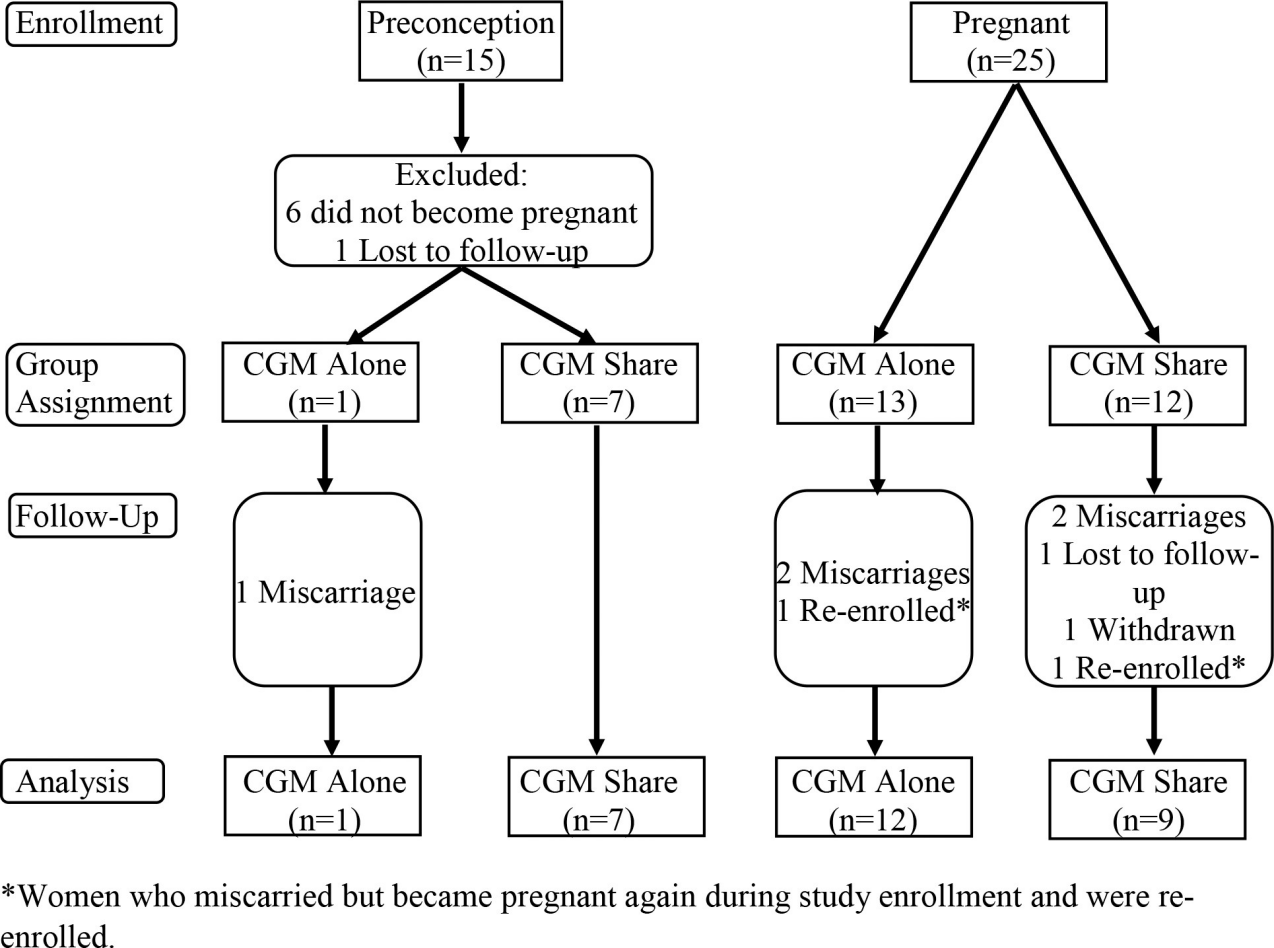
Flow diagram.

Between August 2015 and June 2016, we enrolled 15 women in preconception and 25 during the first trimester. In the preconception group, 8/15 (53%) women became pregnant during the study period. Two women had a miscarriage and became pregnant again during the study period and were re-enrolled. Out of the 35 pregnancies, there were 5 miscarriages, 1 woman dropped, and 1 woman was withdrawn from the study, leaving 28 pregnancies from the prospective cohort for analyses (Fig 1). Thirteen women were assigned to CGM Alone and 15 to CGM Share, there were followers for 12 women in CGM Alone and 14 in CGM Share. No prospectively enrolled women were assigned to the no CGM group. Eight women met eligibility criteria and gave consent for the retrospective cohort (no CGM group).

At preconception baseline, median age, diabetes duration, body mass index, race/ethnicity, insurance coverage, and presence of comorbidities were similar in all 3 groups of pregnant women (Table 1). Significantly more women in the CGM Alone group reported current cigarette use (17% CGM Alone, 0% CGM Share, 38% no CGM, p<0.05) and a history of past cigarette use (67% CGM Alone, 20% CGM Share, 29% no CGM, p<0.05). Initially, more women using CGM Share were on CSII therapy than in the other 2 groups (46% CGM Alone, 87% CGM Share, and 43% no CGM, p<0.05). However, during the pregnancy, 3 women on CGM Alone, 2 on CGM Share, and 3 on no CGM changed from MDI to CSII therapy, thus there was no significant difference in method of insulin delivery during gestation (CSII therapy: 69% CGM Alone, 100% CGM Share, 75% no CGM, p = 0.07). The baseline median HbA1c was 8.1% (7.2–9.0% [25^th^ percentile, 75^th^ percentile]) in women on CGM Alone, 7.1% (6.3–8.4%) in women on CGM Share, and 7.2% (5.5–8.4%) in women not on CGM.

**Table 1:** Baseline characteristics.

Baseline Characteristics	CGM Alone	CGM Share	No CGM	P-Value
Number of participants	13	15	8	
Age (years)^a^	24.4 (21.2, 30.3)	28.9 (26.7, 31.0)	27.6 (20.7, 29.5)	0.243
Diabetes duration (years)^a^	11.6 (6.8, 17.0)	18.0 (10.0, 21.0)	9 (2.0, 15.5)	0.188
Race/ethnicity, n (%)^b^				
Caucasian	8 (62)	14 (93)	--	0.211
Hispanic/Latina	3 (23)	1 (7)	--	
Asian/Oriental	1 (8)	0 (0)	--	
Other	1 (8)	0 (0)	--	
Insurance, n (%)^b^				
Medicaid	6 (46)	4 (27)	--	0.370
Commercial	4 (31)	9 (60)	--	
Other	3 (23)	2 (13)	--	
Body mass index (kg/m^2^)^a^	25.8 (24.6, 28.5)	24.7 (24.2, 31.4)	26.8 (22.6, 33.5)	0.937
Hypertension, n (%)^c^^d^	0 (0)	1 (7)	0 (0)	0.720
Hypercholesterolemia, n (%)^c^^d^	4 (31)	1 (7)	1 (13)	0.122
Cigarette use, n (%)				
Current	2 (17)	0 (0)	3 (38)	0.048
Past	8 (67)	3 (20)	2 (29)	0.039
Method of insulin delivery, n (%)				
MDI	7 (54)	2 (13)	4 (57)	0.041
CSII	6 (46)	13 (87)	3 (43)	
Basal insulin (units)^a^	32.5 (20.0, 54.0)	23.1 (18.6, 30.0)	25.8 (20.8, 30.0)	0.401
Bolus insulin (units)^a^	24.1 (15.5, 31.9)	19.7 (14.3, 28.3)	20.6 (18.1, 25.5)	0.705
Preconception HbA1c (%)^a^	8.1 (7.2, 9.0)	7.1 (6.3, 8.4)	7.2 (5.5, 8.4)	0.202

Abbreviations: HbA1c, hemoglobin A1c; MDI, multiple daily injections; CSII, continuous subcutaneous insulin infusion.

^a^ Median (25^th^ percentile, 75^th^ percentile).

^b^ Data at the time of the pregnancy were not available through retrospective chart review.

^c^ Unknown status for 1 woman on CGM Alone and 1 woman on CGM Share for hypertension, 3 women on CGM Alone and 1 woman on CGM Share for hypercholesterolemia.

^d^ Self-reported.

During the study, there were 3 severe hypoglycemic events in 2 women. Both women were on CGM Alone. One woman was not wearing her CGM at the time of her hypoglycemic event. The other woman was not wearing her CGM during one of her 2 severe hypoglycemic events and during the other event she was alerted to the low sensor glucose by her CGM, took a small correction carbohydrate load, and then lost consciousness. There were no episodes of diabetic ketoacidosis during the study.

**Fig 2:**
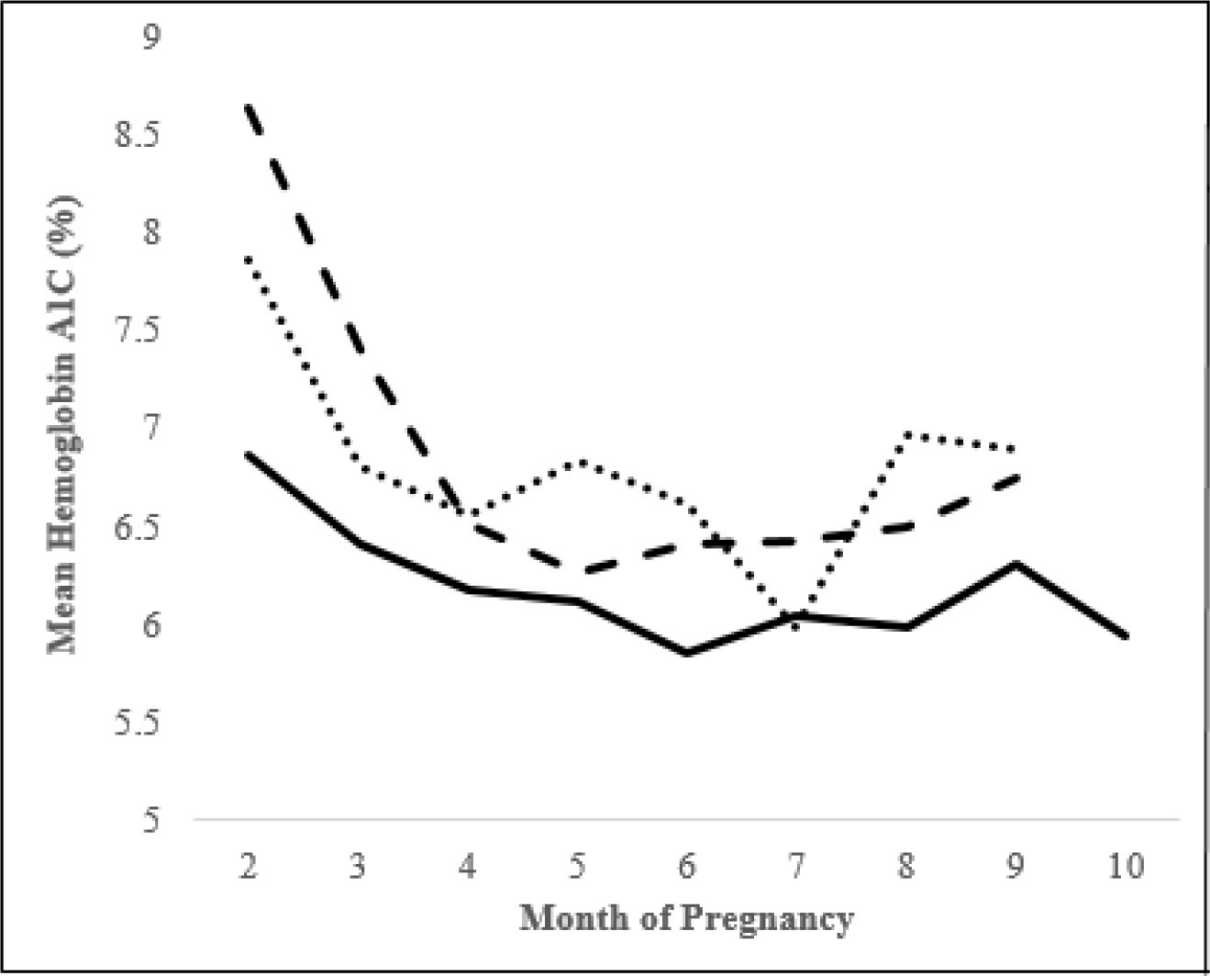
HbA1c throughout gestation. After adjusting for preconception HbA1c, there was a significant difference in changes in HbA1c over time between groups (p = 0.0042). Bold line represents CGM Share group, dashed line represents CGM Alone group, and dotted line represents no CGM group.

HbA1c decreased in all groups as pregnancy progressed and was significantly lower in women using CGM Share compared to CGM Alone or no CGM (p = 0.0042 after adjustment for baseline HbA1c, Fig 2). CGM Share users had a lower median sensor glucose than CGM Alone users, averaged across visits (p = 0.0331, Table 2). CGM Share users spent less time with glucose >180 mg/dL than CGM Alone users, averaged across visits (p = 0.0228, Table 2).

**Table 2:** Maternal CGM measures^a^.

Outcome	1^st^ Trimester		2^nd^ Trimester		3^rd^ Trimester		P-Value
	CGM Alone	CGM Share	CGM Alone	CGM Share	CGM Alone	CGM Share	
Median glucose (mg/dL)	131 (121, 148)	128 (115, 136)	140 (124, 155)	131 (111, 141)	141 (128, 151)	131 (112, 143)	0.0331
Time below target (%) <50 mg/dL <63 mg/dL	1.1 (0.5, 2.0) 7.3 (4.5, 8.9)	1.2 (0.5, 1.5) 7.1 (3.4, 8.2)	0.9 (0.5, 2.2) 4.8 (3.6, 7.4)	1.0 (0.7, 1.8) 5.2 (3.9, 8.1)	0.7 (0.5, 1.3) 4.1 (3.4, 4.4)	0.8 (0.6, 1.0) 4.2 (3.3, 5.9)	0.5704 0.8947
Time in range (%) 63–140 mg/dL	52.9 (46.6, 74.1)	60.0 (54.6, 69.0)	50.7 (44.1, 58.1)	56.8 (48.8, 69.1)	53.4 (42.8, 59.6)	62.4 (50.0, 70.5)	0.0601
Time above target (%) >140 mg/dL >180 mg/dL	38.2 (21.7, 49.0) 17.2 (4.7, 26.6)	35.1 (24.3, 41.0) 14.6 (7.2, 16.4)	46.1 (30.4, 51.7) 20.1(9.9, 30.4)	37.2 (21.9, 43.1) 15.5 (5.3, 22.4)	44.9 (35.4, 52.8) 20.7 (13.3, 27.7)	34.4 (23.1, 46.8) 13.6 (6.5, 19.4)	0.0565 0.0228

Abbreviation: CGM, continuous glucose monitor.

^a^Descriptive statistics are median (25^th^ percentile, 75^th^ percentile) for CGM

The total daily dose (TDD) of insulin per kilogram (kg) of weight was significantly higher in the CGM Alone group compared to CGM Share or no CGM (Alone 0.95±0.03 vs Share 0.79±0.03 vs no CGM 0.77±0.05 units/kg/day, p = 0.0017), even after adjusting for preconception TDD/kg. Maternal weight was higher among CGM Share than CGM Alone (p<0.0001 after adjusting for preconception weight), but gestational weight gain was not different between groups (p>0.05 after adjusting for maternal current smoking at baseline) (Table 3).

**Table 3:** Maternal and fetal outcomes^a^.

Outcome		CGM Alone	CGM Share	P-value
Maternal Outcome	Weight gain (kg) Cesarean section, n (%) Preeclampsia, n (%)	14.1 (10.2, 18.0) 8 (62) 4 (31)	15.5 (12.6, 18.4) 13 (87) 4 (27)	0.3030 0.1413 0.4862
Fetal Outcome	Gestational age (weeks) <37 weeks gestation, n (%) Birth weight (kg) LGA, n (%)^b^ Macrosomia, n (%)^c^ SGA, n (%)^e^ Hypoglycemia, n (%)^f^ Neonatal jaundice, n (%)^f^ Neonatal hypoxemia, n (%) NICU admission, n (%)	35.9 (34.8, 37.0) 7 (54) 3.24 (2.79, 3.69) 5 (39) 0 (0) 0 (0) 7 (58) 8 (67) 6 (46) 5 (39)	37.2 (36.5, 38.0) 4 (27) 3.59 (3.32, 3.85) 8 (53) 3 (20) 1 (7) 11 (73) 5 (33) 1 (7) 7 (47)	0.0629 0.2399 0.2877 0.5147 n/a^d^ n/a^d^ 0.3753 0.1212 n/a^d^ n/a^d^

Abbreviations: kg, kilograms; LGA, large-for-gestational age; NICU, neonatal intensive care unit; SGA, small-for-gestational age.

^a^ Descriptive statistics are median (25^th^ percentile, 75^th^ percentile) and n (%). P-values are adjusted for current maternal smoking at baseline.

^b^ Estimated fetal weight >90^th^ percentile.

^c^ Fetal weight >4 kilograms.

^d^ Model would not converge.

^e^ Estimated fetal weight <10^th^ percentile.

^f^ Data are missing for one woman in the CGM Alone group.

Hospital records from the labor and delivery admission were available for 14 of the 28 pregnancies. Maternal outcomes did not significantly differ between groups (Table 3). Gestational age at delivery was higher in the CGM Share group (35.9 weeks Alone vs 37.2 weeks Share, unadjusted p = 0.0002), birth weight was higher in the CGM Share group (3.24 kg CGM Alone vs 3.59 kg CGM Share infants, p = 0.0429), and neonatal hypoxemia was higher in the CGM Alone group (46% Alone vs 7% Share, unadjusted p = 0.0286). However, there were no differences in gestational age at delivery, infant birth weight, and neonatal hypoxemia after adjusting for baseline maternal smoking (Table 3).

**Fig 3:**
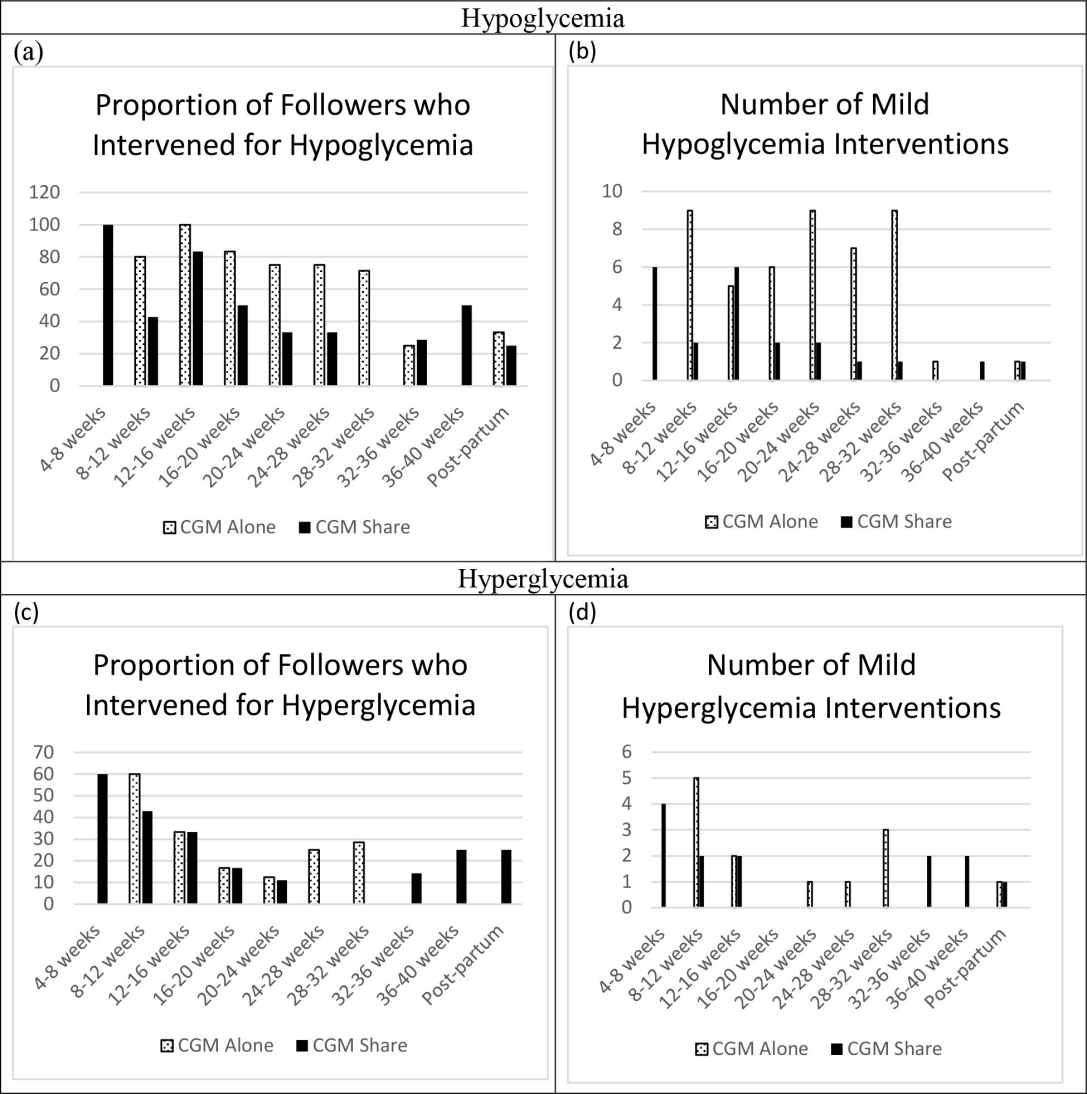
Absolute number of follower interventions. Fig 3A is the proportion of followers who intervened for hypoglycemia on behalf of their pregnant partners. Fig 3B is the number of mild hypoglycemic interventions performed by followers on behalf of their pregnant partners. Fig 3C is the proportion of followers who intervened for hyperglycemia on behalf of their pregnant partners. Fig 3D is the number of mild interventions for hyperglycemia performed by followers on behalf of their pregnant partners.

Followers of CGM Share users reported more alerts for hypoglycemia throughout pregnancy and the early post-partum period. CGM Share followers were more likely to report intervening for hypoglycemia in the first trimester, but were less likely for the remainder of the pregnancy and post-partum (Fig 3A). CGM Alone followers reported 2 episodes of severe hypoglycemia interventions (administration of glucagon or calling emergency services), once at 8–12 weeks and once at 20–24 weeks gestation, while CGM Share followers reported none. CGM Alone followers were more likely to report mild hypoglycemia interventions (giving glucose tablets, giving food or drink, checking a blood glucose level, or calling a healthcare provider) throughout pregnancy but equally likely post-partum compared to CGM Share followers (Fig 3B). Followers of CGM Share users reported more alerts for hyperglycemia throughout pregnancy and early post-partum (Fig 3C). CGM Share followers were more likely to report intervening for hyperglycemia in the first and third trimesters and the early post-partum period, but were less likely in the second trimester (Fig 3D). There were no episodes of severe hyperglycemia interventions (calling emergency services) in either group. CGM Alone followers were more likely to report mild hyperglycemia interventions (giving insulin through a shot or insulin pump, checking a blood glucose level, checking a ketone level, or calling a healthcare provider) in the second trimester, less likely in the first and third trimesters, and equally likely post-partum compared to CGM Share followers.

## Discussion

In this study with 28 pregnancies associated with T1D, we found that CGM use with remote monitoring was associated with a lower HbA1c than CGM use alone or self-monitoring of blood glucose alone. CGM Share users had significantly lower median sensor glucose values and time spent >180 mg/dL across pregnancy than CGM Alone users. Followers reported more alerts for hypoglycemia and hyperglycemia throughout gestation and early post-partum. Followers reported fewer interventions for hypoglycemia with the use of CGM Share compared to CGM Alone. There were no significant differences in gestational health outcomes.

The effect of a family member or friend being able to view CGM trends and get alerts for out-of-range glucose levels with respect to glucose control, fear of hypoglycemia, and health outcomes in pregnant women with T1D has not been previously assessed. Through monthly questionnaires, we found that followers of CGM Share users reported more frequent interventions for hypoglycemia and hyperglycemia for their pregnant partners compared to followers of CGM Alone users. Someone in close contact with a person on CGM Alone is not with her all the time and thus is unlikely to know of glucose levels at the extreme ends of the spectrum when not in her proximity, while a follower of a CGM Share user can access glucose data continuously, including while not in the pregnant partner’s presence. Though we did not inquire how CGM Alone followers were alerted to hypo- and hyperglycemia in their pregnant partners, we asked each pregnant woman to select a main follower with whom she is in close contact. Thus, it is possible that these followers were alerted to low and high glucose levels by recognizing symptoms of hypo- and hyperglycemia in the pregnant women, by asking the pregnant women to check their glucose levels while in their presence or by phone, and/or by identifying patterns of high-risk times for abnormal glucose levels (for example, over night or after meals) and checking on the pregnant women at those times. We previously reported the results of the Hypoglycemia Fear Survey administered to the CGM Alone and CGM Share users in this study and found that mean hypoglycemia total and worry scores were significantly lower among CGM Share users during pregnancy and the early post-partum periods [21]. It is possible that the knowledge of a Share follower itself, the interventions of the Share follower, or a combination of both factors account for the reduced fear of hypoglycemia among our pregnant participants. Another study found a similar result. Litchman and colleagues examined 39 website blog posts with 206 comments about real-time CGM (rtCGM) sharing. Their qualitative analyses revealed multiple themes, one of which was that rtCGM data sharing enhanced patient feelings of safety. The majority of the adults with T1D were interested in the technology for its ability to share hypoglycemia alerts, particularly overnight [22].

One previous study examined remote monitoring in pregnancies associated with T1D. Wojcicki and colleagues randomized 30 pregnant women with T1D to use a glucose meter that uploaded data viewable by a remote diabetologist nightly (study group) or usual care throughout pregnancy. Women in the study group had significantly better glucose control (mean change in mean blood glucose and change in J-index) [13]. In our pilot study of remote monitoring by family/friends, we too found the use of remote monitoring to be associated with some metrics of better glucose control (median sensor glucose and sensor glucose time spent >180 mg/dL). Outside of pregnancy, there was another study that compared alternating nights with remote CGM use (remote) to CGM alone (control) in children and young adults with T1D (n = 57) at diabetes camps [23]. During control nights, participants could hear alarms on their CGM receivers and self-treat for hypoglycemia, while on intervention (remote monitoring) nights only medical personnel received alarms and provided hypoglycemia treatment. Remote monitoring reduced the total number of hypoglycemic events (78 remote vs 119 control nights), the number of hypoglycemic events with glucose <70 mg/dL lasting >1 hour (11 remote vs 33 control, p = 0.003) and >2 hours (0 remote vs 12 control, p = 0.01), the number of hypoglycemic events with glucose <50 mg/dL lasting >30 minutes (0 remote vs 9 control, p = 0.02), and increased the response rate to alarms (100% remote vs 54% control) [23]. We also found a higher number of hypoglycemia interventions with remote monitoring, but only in the first trimester. The duration of remote CGM use may have played a role (many months in a pregnancy study versus a few days in a camp study) and/or the relationship of the viewer of remote data (family/friends in pregnancy versus medical provider in camp study). We additionally found that hyperglycemic interventions were increased in the first trimester in the CGM Share group.

There were baseline differences between the CGM Alone and CGM Share groups which could impact the results. The CGM Share group included 7 women who enrolled preconception compared to 1 in the CGM Alone group. Preconception planning/care is associated with reduced rates of congenital malformations, preterm delivery, perinatal mortality, and maternal hyperglycemia [24–26]. There is evidence that achieving a lower or optimal preconception HbA1c has a particularly large effect on improving gestational outcomes such as rates of congenital anomalies [24–26]. We did adjust for baseline (first trimester) HbA1c levels, but there could be other confounders (such as preconception planning or other measures) that account for some of the differences observed in glycemic control and gestational outcomes in our cohort. Additionally, though the differences were not statistically significantly different between groups, CGM Share users were older, more likely to be Caucasian, had a longer duration of diabetes, higher rates of commercial insurance use, and higher rates of insulin pump use at baseline. Had the sample size been larger, it is possible that some or all of these dissimilarities at baseline would have been statistically significantly different between groups because the groups were stratified, not randomized, based on personal device ownership of Apple products. These group differences may also independently influence glucose control and gestational outcomes beyond what would be seen from the intervention alone.

This study has several strengths. The same device was used in both CGM groups, the rates of CGM use were high, well-validated measures of fear of hypoglycemia were collected prospectively in the CGM groups, and followers for both CGM groups were surveyed monthly. Our study was limited in that the data from the no CGM group were retrospective, as a pilot study the numbers of participants in the prospective groups were small, there were no baseline CGM data prior to group assignments, and it was not randomized because providing compatible devices for remote data viewing to all CGM Share users was cost prohibitive and thus they were self-selected. It’s possible that the characteristics associated with device compatibility at baseline (such as financial access) are themselves associated with changes in health behaviors and outcomes.

## Conclusions

Pregnancy is a time when women with diabetes are often more vigilant about optimizing glucose management, engaging in good diabetes self-care behaviors, and motivated to improve health outcomes. However, they often concomitantly experience increased stress, emotional burden, and hypoglycemic events in their attempts to improve diabetes control. Remote monitoring may enhance engagement opportunities for their loved ones and supporters to assist them throughout gestation. These preliminary, pilot data show promise for a potential role of CGM therapy with remote monitoring to improve clinical care in pregnant women with T1D through a broader support system for women involving family/friends. The results from this study should be considered with caution as the stratification to study group assignment did yield groups that were inherently different at baseline, which could affect the outcome measures. More studies, especially randomized controlled trials, are needed to corroborate these findings and describe how remote monitoring may affect maternal glycemic control.

## Supporting information

S1 Checklist Trend statement checklist. (PDF)

S1 Data Investigator-initiated pilot prospective CGM Quality Improvement (QI) project investigator initiated study protocol. (PDF)

S1 File Pilot CGM project in pregnancy. (PDF)
