# Supplementary material for: Continuous glucose monitor use with and without remote monitoring in pregnant women with type 1 diabetes: A pilot study
Source: PLoS One. 2020 Apr 16;15(4):e0230476. doi: 10.1371/journal.pone.0230476 (PMC7162510; doi:10.1371/journal.pone.0230476)
Supplement: S1 Data — (PDF) [file pone.0230476.s002.pdf]

# Investigator-initiated Pilot Prospective CGM Quality Improvement (QI) Project

## Investigator Initiated Study Protocol

| | |
|---|---|
| **Protocol #:** | **WIRB # 20151413, COMIRB #15-1006** |
| **Principal Investigator:** | **Sarit Polsky, MD, MPH**<br>Assistant Professor of Medicine and Pediatrics |
| **Co-Investigators:** | Satish Garg, MD<br>Professor of Medicine and Pediatrics |
| | Rachel Garcetti, PA<br>Instructor of Pediatrics and Medicine |
| | Mary Voelmle, NP, CDE<br>Instructor of Pediatrics and Medicine |
| **Chief Coordinators:** | Mara Schwartz RN, Daniel Ruck, CDE, Lisa Meyers, CDE, Terra Thompson, RN |
| **Co-Coordinators**: | Dominique Giordano, PRA, Ashleigh Downs, PRA, Scott Brackett, PRA |
| **Primary Institution:** | Barbara Davis Center for Diabetes<br>University of Colorado Denver<br>1775 Aurora Court, Mail Stop A140<br>Aurora, CO 80045 |
| **Sponsor:** | DexCom, Inc. |

## Abbreviations:

| | |
|---|---|
| **A1C** | **Hemoglobin A1C** |
| **ADA** | **American Diabetes Association** |
| **AE** | **Adverse Event** |
| **ATTD** | **Advanced Technologies and Treatments for Diabetes** |
| **CDE** | **Certified Diabetes Educator** |
| **CGM** | **Continuous Glucose Monitoring** |
| **CSII** | **Continuous Subcutaneous Insulin Infusion** |
| **DKA** | **Diabetic Ketoacidosis** |
| **FDA** | **Food and Drug Administration** |
| **HBGI** | **High Blood Glucose Index** |
| **LBGI** | **Low Blood Glucose Index** |
| **MAGE** | **Mean Amplitude of Glycemic Excursions** |
| **MDI** | **Multiple Daily Injections** |
| **SAE** | **Serious Adverse Event** |
| **SMBG** | **Self-Monitoring of Blood Glucose** |
| **T1DM** | **Type 1 Diabetes** |

**Project Overview/Summary:**

This is a single-center, prospective, 'open-label,' investigator-initiated pilot study evaluating the role of continuous glucose monitor (CGM) use either alone or with remote monitoring capabilities that enable subjects to share CGM data with family and friends (whom we will call "followers" in this protocol) among women with T1DM associated with pregnancy. We hypothesize that CGM use will decrease percentage of glucose values below the target range, decrease frequency of severe hypoglycemic episodes, and increase post-prandial glucose values into the target range, with a possible reduction in A1C compared to not using CGM during pregnancy. We hypothesize that the use of remote technology **(Share™ technology)** with subject's designated follower will decrease hypoglycemia fear and the frequency of severe hypoglycemic episodes compared to CGM use alone in pregnancy or not using CGM use in pregnancy. This study aims to improve maternal glucose management among women with T1DM with a hope to also improve health outcomes during pregnancy.

The study will enroll up to 40 patients from the Barbara Davis Center for Childhood Diabetes (BDC) Adult clinic over the age of 18 years who are pregnant (13 weeks gestation or less) or plan to become pregnant within the following 6 months. The study will also attempt to enroll up to 40 followers (one per pregnant subject) who are family members or friends of the pregnant subjects and over the age of 18 years. All subjects meeting these criteria will be invited to sign informed consent and participate in the study. All pregnant subjects will be stratified into one of three groups: (1) CGM use alone, (2) CGM use with Share™, (3) no CGM use. CGM use will be offered to all subjects, but individuals who do not want to wear a CGM will be stratified to Group 3. Stratification for Groups 1 and 2 will depend on the following: subjects and followers who have iPhones, iPads, and/or iPod touches will be placed into Group 2 which requires one of these devices to function, while all others will be placed in Group 1. All subjects will be followed for study visits with similar frequency during the pregnancy, and subjects in Groups 1 and 2 will wear the Dexcom G4 Platinum® CGM system from the first pregnancy visit until delivery. All subjects in Group 2 will also use the Share™ technology from the first pregnancy visit until delivery.

CGM data will be analyzed for mean blood glucose values, time spent in hyperglycemic (>240, >300mg/dl) and hypoglycemic (<50, <70, <80 mg/dl) ranges and various indices of glycemic variability. Laboratory analysis for A1C will be performed at baseline, monthly thereafter during the pregnancy, and post-partum. Routine blood tests will be performed in all subjects in each trimester (see Table 1).

**Primary outcomes** will be:
1) Glucose variability as measured by glucose excursions from self-monitoring blood glucose (SMBG) data through log books and downloads as well as CGM downloads and various indices [J-index, mean amplitude of glycemic excursion (MAGE), high blood glucose index (HBGI), low blood glucose index (LBGI)] from CGM downloads.
2) Changes detected by the hypoglycemia fear questionnaire.
.
**Secondary outcomes** will be:
1) How CGM alone and CGM with Share™ usage affect A1C compared to no CGM use,
2) Progression of diabetes complications (retinopathy, nephropathy),
3) Maternal and fetal outcomes (eclampsia/pre-eclampsia, live birth rates, birth weight, neonatal hypoglycemia, and other similar measures).

**Introduction:**

Among the US population, 29.1 million people (9.3%) have diabetes (1). Pre-existing diabetes has to be managed very carefully throughout pregnancy to optimize maternal and fetal outcomes (2). Among women

with T1DM who are pregnant, the tools that we have to regulate maternal glucose control include multiple daily injections (MDI), continuous subcutaneous insulin infusion therapy (CSII) also known as insulin pump therapy, and for high-risk individuals continuous glucose monitoring (CGM) (2). CGM use in pregnancy has been shown to be safe, to reduce the frequency of severe hypoglycemia, and to improve A1C values (3-6). Sensor-augmented pump therapy with CGM use continuously was shown to lower A1C more than intermittent use in pregnant women with T1DM (7). However, few studies have measured changes in glucose variability that occur with CGM use during pregnancy and no studies have evaluated how tools that allow family and friends (followers) to access glucose trends affect outcomes during pregnancy.

The goal of this prospective, non-randomized, pilot quality improvement study is to evaluate how CGM usage with and without remote monitoring capabilities affects glucose variability, A1C control, and patient-related outcomes compared to non-CGM use in pregnancy among women with T1DM. This study may lead to further investigations about the role of mobile phones and handheld devices (such as iPod touch) for T1DM self-care in pregnancy.

The Barbara Davis Center (BDC) Adult Clinic follows ~3,000 patients with T1DM. Sarit Polsky, MD, MPH is this study's Principal Investigator and the director of the Pregnancy and Women's Health Clinic at the BDC, which includes an extensive multidisciplinary team (physician's assistant, nurse practitioner, registered nurses, certified diabetes educators, registered dieticians, and a social worker). The BDC Pregnancy and Women's Health Clinic has all the necessary resources to perform such a trial. Moreover, the investigators have extensive experience conducting clinical trials, including trials during pregnancy.

**Primary Objectives:**

The primary objectives of this pilot study are to improve maternal glucose management, to assess the role of CGM usage either alone or with remote monitoring capabilities (Share™) and changes detected by the hypoglycemia fear questionnaire among women with T1DM associated with pregnancy.

**Secondary objectives:**

1. Assessment of remote monitoring capabilities of followers (Share™ usage) that lead to interventions for hypoglycemia and hyperglycemia that affect subjects wearing CGM.
2. Reduction in A1C from baseline to 3 months with maintained control at 6 and 9 months.
3. Assess progression or stability of diabetes complications (retinopathy, nephropathy).
4. Assess effects of CGM usage on maternal and fetal health outcomes (eclampsia/pre-eclampsia, live birth rates, birth weight, neonatal hypoglycemia, and other similar measures).

**Research Design:**

**What do we intend to do?**

We intend to execute a single-center, prospective, non-randomized, 'open-label,' investigator initiated pilot study evaluating the effect of CGM usage with or without remote monitoring capabilities on women with T1D throughout pregnancy and compare outcomes to women who do not use a CGM during pregnancy. We also intend to ask a main follower for each woman about his/her knowledge of extreme glucose values among subjects and interventions on a subject's behalf. See Figure 1 for study design.

**How are we going to do the work?**

The BDC is primarily a T1DM clinical and research center and follows large numbers of patients with T1DM. Pregnant women are followed in the Pregnancy and Women's Health clinic at the BDC. All site personnel will attend an investigator's meeting, which will be organized and conducted before trial initiation. All subjects prospectively enrolled will sign an informed consent form approved by the Western Institutional Review Board (WIRB) through the Colorado Multiple Institutional Review Board (COMIRB). A follower, designated by each subject, will also be asked to sign an informed consent form approved by the Western Institutional Review Board (WIRB) through the Colorado Multiple Institutional Review Board (COMIRB), however follower consent will not obligatory for a pregnant subject to participate in the study. Subjects identified through the retrospective chart review of existing data who are no longer pregnant, may have irregular visits to the Barbara Davis Center and in some cases only return to clinic once per year. As such, these subjects will be contacted by phone or e-mail to see if they are interested in participating in the study. Interested post-partum women will be sent the consent form approved by WIRB through the COMIRB prior to the informed consent discussion. We will undergo consenting procedures with subjects in person, when possible, or by phone and will have subjects sign the consent form and send it back to us. After our staff signs the consent form, subjects will receive a copy of the completed, signed consent form. Communications with the consent form with subjects in the retrospective chart review will take place via e-mail, United States Postal Service, or fax (to be determined by availability, security of devices, and subject preference).

**What is the study population?**

The primary study population will consist of women at the BDC in the pre-conception and early gestational periods that are eligible for this pilot investigator-initiated CGM QI Project. Subjects will be women intending to become pregnant or within the first 13 weeks of gestation, 18 years of age or older, using MDI or CSII therapy, with any A1C level, and willing to participate in the study. There is also a no CGM comparison group of women who were pregnant around the time study enrollment began and earlier who were patients at the BDC and whose data are available retrospectively through the electronic medical record system. The secondary study population will be designated followers of pregnant patients at the BDC. Followers will be family members or friends of pregnant subjects who are willing to participate in the study.

**How are subjects being recruited into the study?**

Subjects will be recruited from the BDC Pregnancy and Women's Health clinic for screening, as well as the BDC Adult Clinic. During pre-conception counseling visits, women will be approached about participating in the study (if eligible). We will maintain a list of women intending to conceive within the next 6 months, ask women to come in for a pregnancy visit as soon as pregnancy is confirmed, and stratify women into 1 of 3 groups described below. If after 6 months a woman on the list has not yet come in for a pregnancy visit, we will contact her to inquire if she still plans to conceive (keep subject on the list) or not (remove from list).

Women who have their first pregnancy visit at the BDC at 13 weeks of gestation or less will be screened for the study. If eligible and interested in participating, after signing the informed consent, women will be stratified to either CGM use alone (Group 1) or CGM use with Share™ (Group 2). As Share™ requires specific device use, Group 1 subjects will be women who do not have an iPhone, iPad, or iPod touch or whose main follower does not have an iPhone, iPad, or iPod touch. Group 2 subjects will be women who have an iPhone, iPad, or iPod touch and whose main follower also has an iPhone, iPad, iPod touch, or other compatible device for downloading the Follower app. If eligible to participate but not interested in using a Dexcom CGM, women will be invited to sign informed consent so that we may follow their

outcomes over the course of the pregnancy while diabetes control is managed per usual care (Group 1 for women using a non-Dexcom CGM such as Medtronic Enlite CGM or Group 3 for no CGM use). A PRA will maintain an active list of individuals and will update the list continuously as women are assigned into groups. All subjects will have similar clinical visits throughout the pregnancy.

Subjects stratified to Group 2, CGM use with Share™, will be asked to designate followers who will be able to view glucose trends and receive alerts for high or low sensor glucose values on an iPhone, iPad, iPod touch, and/or other compatible device. While multiple followers are allowed, one will be the main follower and will be invited to sign informed consent at the screening visit or within the first two months of each subject's study participation. Subjects stratified into Groups 1 and 3 will be asked to designate a main follower who may be aware of a subject's extreme glucose values (lows or highs) through contact with her and who may intervene on her behalf in such cases and will be invited to sign informed consent at the screening visit or within the first two months of each subject's study participation.

**What are the treatment groups?**

There will be three 'open-label' treatment arms consisting of routine care in the Pregnancy and Women's Health clinic at the BDC (no CGM use), an intervention arm using the Dexcom G4 or G5 Platinum® CGM system during pregnancy, and an additional treatment arm using the Dexcom G4 or G5 Platinum® CGM system with Share™ remote monitoring capabilities during pregnancy. As this is a non-randomized study and most women will opt to be in the Dexcom alone or Dexcom Share groups, we will include pregnant no CGM users identified through a retrospective chart review into the no CGM arm of the study.

**How many subjects per treatment arm?**

A total of up to 40 pregnant women will be prospectively recruited into the study. As this is an 'open-label' study whose group stratification is based on subject characteristics (willingness to wear CGM, subject possession of iPhone, iPad, or iPod touch, follower possession of iPhone, iPad, or iPod touch), we cannot predict the number of subjects per treatment arm. Based on our current pregnant population's willingness to use CGM and device usage, we anticipate that 10 subjects will be in Group 1, 20 subjects in Group 2, and 10 subjects in Group 3. We will include up to 30 additional women in Group 3 identified through a retrospective chart review who were pregnant around the time of study enrollment and before the study began.

In addition, there will one main follower for each prospectively enrolled pregnant subject (up to 40 adults) who will be asked questions once per month about knowledge of the subject's glucose trends and interventions that s/he may have done on her behalf over the last month.

**What is the schedule of events for the treatment groups?**

After screening, when pregnancy is confirmed subjects will be stratified to Group 1, 2, or 3. Subjects in all groups will have similar study/clinic visits for data acquisition at the screening, pregnancy visits (which occur a minimum of monthly throughout gestation), and post-partum clinic visit. All subjects will be required to test blood glucoses at least 7 times a day throughout pregnancy. All subjects will be asked to log the SMBG values 7 times a day with insulin doses for meals for 1 week every trimester of the pregnancy.

Subjects stratified to CGM wear will be provided with a Dexcom G4 CGM, transmitter, and sensors (4 per month). Subjects stratified to CGM wear who want to use their personal Dexcom G5 devices will be asked to provider Dexcom mobile usernames (if they use G5 with an iPhone) and will be provided with sensors (4

per month). Subjects already using a Dexcom G4 or G5 CGM will be provided only with the sensors. Replacement transmitters will be provided on an as needed basis during the study. A designated CDE or nurse will train each subject in the use of CGM at Visit 2, including how to use the sensor data (rate of change of glucose, to cross-check with fingerstick blood glucose as per FDA label before action is taken). Subjects in Group 2 will be additionally instructed on how to use the Platinum System with Share™ and will set up contact lists with family and friends at that visit. Subjects will be asked to wear the CGM for the duration of the pregnancy and to change sensor sites every 7 days. All subjects will be encouraged to check the blood glucose level (SMBG) before taking action on the CGM glucose values (as per the FDA label). Subjects in Groups 1 through 3 will be asked to designate a main follower who is willing to sign informed consent and fill out a questionnaire once a month.

Subjects will be asked to return to clinic a minimum of once per month for pregnancy care with a BDC provider and to receive the next month's sensors from a designated PRA. There are women whom we ask to return to clinic based on their clinical needs every week or every 2 weeks for pregnancy care who will be allowed to pick up sensors on a biweekly basis rather than monthly, if preferred. If a patient misses an appointment and needs to pick up sensors, she will be asked to reschedule her pregnancy visit and will be allowed to pick up sensors from the PRA for one more month. All subjects will be allowed to keep the devices used in the study upon completion.

The final study visit will occur in conjunction with the post-partum visit.

**What is the schedule of events for the follower group?**

After screening, subjects assigned to Group 2 will be asked to designate a main follower and up to 4 other followers who will be allowed access to view glucose trends, to receive low sensor glucose alerts, and to receive high sensor glucose alerts for the subject. After screening, subjects assigned to Groups 1 or 3 will be asked to designate a main follower with whom she shares time and/or information about her diabetes care and who may intervene on her behalf should a need arise (e.g., provide glucagon for severe hypoglycemia, or suggest going to the hospital for extreme hyperglycemia). Main followers who sign informed consent, which will be obtained in person or by phone, will be asked to fill out a brief questionnaire once per month inquiring about the frequency of viewing information about glucose trends, receiving alerts or information about extreme low and high glucose values, and acting upon aforementioned data to assist the subject. The main follower can fill out the Follower Questionnaire on paper or s/he can receive the follower questionnaire electronically through REDCap is s/he has a working e-mail address (see *Data Collection Tools* section for details).

**Description of Mobile Technology:**

Subjects in Group 2 will be trained on the use of Dexcom G4 Platinum® CGM system with Share™. This is a bluetooth low energy secure wireless communication system that allows remote viewing of sensor glucose levels, trends, and data between the person with diabetes wearing the CGM and her designated family members or friends. The followers can additionally receive alerts (alarms) for pre-specified low sensor glucose and high sensor glucose values. The person with diabetes can also designate herself as a follower to allow access to data through the approved devices. This technology is currently only available for use with Apple iPhone®, iPad, or iPod touch®. Followers have to have access to a device (smart phone) that allows download of the Dexcom Follower app. We will not provide iPhones, iPads, or iPods to subjects or followers for this study.

**Visit Descriptions:**

**Screening, Visit 1 (Week 0):**

The study will begin with a screening visit either during a pre-conception counseling visit or within the first 13 weeks of gestation: Visit 1, (Week - 0). All subjects meeting inclusion/exclusion criteria will be invited to sign informed consent, which will be obtained at this study visit.

During this visit, pregnant subjects will receive a physical exam, vitals, medical history, demographics, and concomitant medications. Laboratory tests will be performed to assess for A1C. SMBG values will be downloaded. If applicable CGM units and insulin pumps will be downloaded.

**Stratification, Visit 2 (Week 1):**

Subjects enrolled in the study from pre-conception will be asked to come to clinic as soon as a home pregnancy test or serum blood test confirms pregnancy, during which time routine clinical care will be provided and subjects will be stratified to 1 of 3 study arms based on subject characteristics. Subjects who did not have a pre-conception counseling visit who come to clinic for routine clinical care before 13 weeks of gestation will combine Visit 1 (screening) with Visit 2 (stratification with CGM start). A urine pregnancy test will be administered to confirm pregnancy. Per routine clinical care, we will screen for diabetic neuropathy on physical exam, thyroid disease (TSH blood draw), and kidney disease (24-hour urine protein with creatinine). These tests will be repeated once each trimester. Also per routine clinical care, women will be asked to get a dilated retinal eye exam each trimester and to provide the BDC with a copy of the provider's report of each exam.

Women stratified to Groups 1 and 2 who own a Dexcom G4 or G5 Platinum CGM® will be given 4 sensors. Women stratified to Groups 1 and 2 who do not own a Dexcom G4 or G5 Platinum CGM® will be given Dexcom G4 CGM units, transmitters, and 4 sensors. They will also receive training in the insertion of sensors and CGM use. A Dexcom G4 Platinum® CGM sensor will be inserted that day and will be worn in 7 day periods throughout the study. Although subjects will be asked to wear the device 7 days/week, satisfactory data capture rate is regarded as 5 days of successful CGM readings. The CGM data will be unblinded (real-time use) such that subjects will be able to view glucose values and trends, as well as receive alerts for low and high sensor glucose values. Women will be asked to check SMBG at least 7 times daily (fasting, before meals, 2 hours after a meals, and at bedtime) for the duration of the study.

Women in Group 2 will be trained in the use of the Share™ technology. They will be asked to designate a main follower and up to 4 additional followers who will receive access to sensor glucose data remotely through the secure, wireless communication system. Recommendations will be made for low and high glucose sensor alerts that will be individualized to each subject's needs (for example a low glucose alert with a higher glucose value for subjects with hypoglycemia unawareness compared to those without it). The main follower for each subject will be approached and asked to sign informed consent at Visit 2 or within 2 months of the subject's first pregnancy visit. Study participation of a main follower will be encouraged but not obligatory.

Women unwilling to wear a CGM but willing to participate in the study (Group 3) will be asked to check SMBG at least 7 times daily (fasting, before each meal, and 2 hours after meals, and at bedtime), per standard of care, for the duration of the study. All 3 groups will receive education about diabetes during pregnancy.

Women in Groups 1 and 3 will be asked to designate a main follower who will not receive data through a device, but rather through contact with the subject. The main follower for each subject (Groups 1 through 3)

will be approached and asked to sign informed consent at Visit 2 or within 2 months of the subject's first pregnancy visit. Study participation of a main follower will be encouraged but not obligatory.

Data on all subjects for medical history, existing diabetes complications, medications, vital signs, height/weight, A1C (current and most-immediately pre-conception), insulin doses, glucometer downloads, insulin pump downloads (if applicable), CGM downloads (if applicable), and adverse events (AEs) will be collected. If the woman was seen at the Barbara Davis Center prior to the consent visit, we will do a retrospective chart review for medications, vital signs, height/weight, A1C, insulin doses, glucometer downloads, insulin pump downloads (if applicable), and CGM downloads (if applicable) for the most-immediate pre-conception clinic visit and for visits during the current pregnancy. Each subject will be given a hypoglycemic fear questionnaire. We will ask women about interventions that have taken place over the last month for extremely low and high glucose values, directed by patient or a third party, and keep the information on research source documents.

**Visits 3 through 13 (Weeks 5 to 40):**

Subjects will come to the BDC for routine clinical care at least once per month for the duration of the pregnancy (through gestational week 40, though some women deliver up to 42 weeks gestation) and for continued study participation. Per routine clinical care, we will screen for diabetic neuropathy on physical exam, thyroid disease (TSH blood draw), and kidney disease (24-hour urine protein with creatinine) at least once each trimester. Also per routine clinical care, women will be asked to get a dilated retinal eye exam each trimester and to provide the BDC with a copy of the provider's report of each exam.

Subjects in Groups 1 and 2 will receive 4 new sensors per month, but may choose to get 2 sensors twice a month instead, at study visits. Use of acetaminophen-containing medications like Tylenol® during sensor wear may affect device performance, therefore we will ask subjects to keep a log of each time these medications are used. We will ask subjects at each monthly visit whether she used acetaminophen-containing medications during the previous month and when. Followers will be administered a brief questionnaire at the beginning of every month inquiring on the frequency of low alerts, high alerts, viewing glucose trends, and actions they may have taken on behalf of the subjects because of the above (see Table 2 and *Follower Questionnaire* for details). On source documents, we will also record how often subjects required an intervention for low or high glucose values within the previous month, and the nature of the interventions. If in Group 2, we will keep source documentation about interventions resulting from a Share™ alert within the previous month.

Data on all pregnant subjects for medications, vital signs, height/weight, insulin doses, glucometer downloads, insulin pump downloads (if applicable), and CGM downloads (if applicable) will be collected at least once per month and as often as every week. All subjects will be asked to collect a 7-point profile for SMBG measurements (blood glucose measurements before meals, 2 hours after meals, and at bedtime) along with meal insulin dosing for 1 week once per trimester (see SMBG Questionnaire). A1C will be measured once per month. Subjects will be asked about updated concomitant drugs and any AEs that may have occurred. Each subject will be given a hypoglycemic fear questionnaire.

**Visit 14 (Weeks 44 to 52):**

All subjects will have routine post-partum clinical care between 4 and 6 weeks post-partum. However, a post-partum visit up to 12 weeks post-partum will be accepted as a final study visit. Data on all subjects for medications, vital signs, height/weight, insulin doses, SMBG values, glucometer downloads, insulin pump downloads (if applicable), and CGM downloads (if applicable) will be collected. All subjects will be asked to

collect a 7-point profile for SMBG measurements (blood glucose measurements before meals, 2 hours after meals, and at bedtime) along with meal insulin dosing for 1 week in the post-partum period (see SMBG Questionnaire). A1C will be measured. Subjects will be asked about updated concomitant drugs and any AEs that may have occurred. Subjects in Groups 1 and 2 may have had a Dexcom G4 Platinum CGM® for personal use prior to study participation and may thereafter still wear the device after labor and delivery. In such cases, CGM downloads will be included in study data. On source documents, we will record how often subjects required an intervention for low or high glucose values within the previous month, and the nature of the interventions. Subjects will be administered a questionnaire inquiring about the labor and delivery process, maternal and fetal complications before and after delivery, baby's anthropometrics at birth (weight and length), baby's current age, breastfeeding, and other measures (see *BDC Pregnancy CGM Post-Partum Questionnaire* for details). If the pregnancy ended early (for example, spontaneous abortion or stillbirth) or resulted in fetal or neonatal death (for example, stillbirth) we will obtain data about these outcomes. Each subject will be given a hypoglycemic fear questionnaire. Followers will be asked to fill out their last *Follower Questionnaire* during the post-partum period.

**Control Arm:**

As outlined in the proposal, the subjects prospectively enrolled in this arm (Group 3) will receive the same care as in the intervention arms (Groups 1 and 2) with the exception of not having access to the Dexcom G4 Platinum CGM® with or without Share™ technology**.**

There will also be a control arm of pregnant patients at the BDC identified through a retrospective chart review who were pregnant around the time of study enrollment or before and who did not use a CGM device throughout their pregnancies. The retrospective chart review will collect data on a subject's medical history, diabetes complications, medications, vital signs, height/weight, A1C, insulin doses, glucose meter downloads, insulin pump downloads (if applicable), and CGM downloads (if applicable) for the most immediate pre-conception visit, all pregnancy visits, and the post-partum visit. Subjects identified through the retrospective chart review will not have filled out any of our questionnaires (baseline, hypoglycemic fear, and postpartum questionnaires), nor will they have had followers who filled out questionnaires. They will not be asked to fill out these questionnaires.

**Inclusion Criteria:**

Subjects that meet the following criteria will be considered for admission to the study:

1. Signed informed consent before any study-related activities
2. Female aged 18 years and older with T1D duration >1 year
3. Pregnancy with confirmation of gestational age 13 weeks or less
4. Willingness to routinely practice at least 3-7 blood glucose measurements per day
5. Using MDI or CSII therapy
6. Any A1C level
7. Ability and willingness to adhere to the protocol including scheduled study visits for the duration of the pregnancy
8. Able to speak, read, and write English

**Exclusion Criteria:**

Subjects will be excluded from the study if any of the following apply:

1. Unwilling to sign informed consent and participate in the study
2. Female age 17 years or younger
3. T1D duration <1 year
4. Pregnancy in gestational week 14 or higher
5. Extensive skin changes/diseases that inhibit wearing a sensor on normal skin
6. Severe known allergy to adhesives within the last 3 months
7. Any other condition, as determined by the investigator, which could make the subject unsuitable for the trial, impairs the subject's suitability for the trial, or impairs the validity of the informed consent

**Risks and Justification of Procedures:**

**CGM:** Continuous glucose monitoring is an FDA approved technology that offers a unique and practical way of identifying and differentiating clinical benefits of intensive diabetes care. The American Diabetes Association currently recommends CGM use during pregnancy for high-risk individuals (2). We currently have hundreds of patients, which include many pregnant patients, in the Adult Clinic at The BDC using CGM on a regular basis as part of usual medical management of their diabetes. Subjects may experience pain at the site of sensor insertion. The adhesive pads for the sensor may cause skin erythema for 1 to 2 days or more. An allergic reaction to 1 or more parts of CGM devices may occur which can be mild, moderate, or severe (rare). In rare cases, an infection at the sensor site may occur. In rare cases, the sensor or needle may break inside the body and would require a minor surgical procedure to remove it.

**Data Collection Tools:**

A confidential subject database will be established to maintain study data. Data will be entered into REDCap (Research Electronic Data Capture). REDCap is an internal secure, computerized database system at the University of Colorado Denver. This system allows data entry, survey/questionnaire building, data exportation to statistical packages, and is HIPAA compliant. Each subject will be assigned an identification number, which will be used to code and identify all of that subject's records. This will avoid the continual use of subject names. REDCap surveys can be sent to study participants via e-mail for direct input into the database. The electronic medical record system at the Barbara Davis Center will be used to conduct a retrospective chart review to identify BDC patients not using a CGM device who were pregnant around the time of study enrollment and before. Their data will be put into REDCap and will be de-indentified for data analyses. All study data will be locked in PI's office and all relevant computer study files will be input on staff computers, which are password protected and contain encryption software. Data storage will be take place on a secured server maintained by the University of Colorado. The server is backed up nightly and a copy of the back-up file is kept off site in a secure facility. Data access will be limited to study personnel. Study results may be presented in the form of posters, abstracts, oral presentations, or publications at academic meetings or in journals. In all forms of study result reporting, subject identification will not be disclosed. A study subject may access his/her protected health information at any time by requesting said information in writing of the investigator. The investigative team has been trained in IRB and HIPAA compliance issues and will maintain confidentiality and protect health information. The above-stated procedures have been highly effective in preventing breaches of patient confidentiality for the prior and current research studies in which the PI has been and continues to be involved at the University of Colorado Denver.

**Safety Measures:**

A data safety monitoring board (DSMB) consisting of Drs. Janet Snell-Bergeon (Epidemiologist and Statistician), Peter Gottlieb, and Viral Shah will oversee safety data throughout the study via reporting of hypoglycemic events, adverse events (AE), and severe adverse events (SAEs) if any.

All AEs, reported spontaneously by the subject, as well as those noted by the investigator or study staff, regardless of seriousness, severity or expectedness will be recorded on source documents from the time of obtaining the informed consent.

Severe hypoglycemia as defined as a hypoglycemic episode requiring assistance or hospitalization with or without an SMBG is to be reported as an SAE. A hypoglycemic event “requiring assistance” is determined when the subject is unable to treat the event on her own. Any untoward event resulting in hospitalization, extension of hospitalization, death, or threat of death will also be considered an SAE. SAEs will be reported to the DSMB. The endpoints of A1C and mean glucose readings will be excluded from SAE reporting.

**Weakness of the Study**

This is not a randomized clinical trial, as the Dexcom remote monitoring technology is not available for other types of handheld devices besides the iPhone, iPad, and iPod Touch. This is a pilot study as no one has previously evaluated the role of this mobile technology in diabetes care complicated by pregnancy. Our goal is to learn from this study and plan for a much larger study where handheld devices are also provided and thus randomization can occur in the future after implementing changes that will be learned from this pilot study.

**Statistical Analysis Plan:**

Statistical analysis is expected to be completed within 6 months of the last study visit of the last enrolled subject. Analysis will be conducted with an intention to treat approach. We will aim to recruit up to 40 pregnant subjects and plan for a 20% drop-out rate (n=8) to have 32 subjects complete the study. We aim to enroll up to 40 followers, but as their participation will be not be obligatory outcomes will not depend on their enrollment. As this is a pilot-study and has no formal power, calculations will be performed for the primary or secondary endpoints.

The primary endpoints are glucose variability and changes detected by hypoglycemic fear survey. We will measure indices of glucose variability (mean glucose ± SD, % of glucose within/above/below target ranges, J Index , High Blood Glucose Index [HBGI], Low Blood Glucose Index [LBGI], Mean Amplitude of Glycemic Excursions [MAGE], $CONGA_n$) throughout pregnancy using the SMBG 7-point profiles for one week taken once per trimester. Hypoglycemic fear survey results will be assessed once monthly over the pregnancy. We will compare outcomes within individuals over time and between individuals of the different study arms. We will test whether outcomes differ between women using Dexcom alone and women using Dexcom with Share™ using interaction terms, and will stratify analyses if differences by group are greater than 10 mg/dL in mean glucose levels are detected. Since SMBG logs will be taken at multiple time points over the pregnancy and since CGMs will be worn continuously for the duration of each pregnancy, linear mixed models will be used to assess changes in outcome measures (GV, time spent in target below or above target) over time in each trimester of pregnancy. We will use linear mixed models to assess changes in the hypoglycemic fear survey in each month of the pregnancy. Secondary endpoints include A1C differences among study arms. Improvement in glycemic variability parameters with a possible reduction of A1C by 0.3%, measured as the difference of the mean A1C laboratory values of the arms of the study between baseline (0-3 months, first trimester), second trimester (3-6 months), and third trimester (6 to 9 months). Mixed models account for missing data, allow for an unstructured covariance matrix for repeated measures, and account for inter-subjects correlation of measurements. Secondary outcomes will be used

in the same mixed models. Differences in frequency of low alert glucose rates, high alert glucose rates, and interventions for low or high glucose values among the followers for each study arm will be analyzed with student t-tests. A p-value $< 0.05$ will be considered significant, and we will perform statistical analyses with SAS software (version 9.3, SAS Institute, Cary NC).

**Publication Strategy:**

The BDC is a leading clinical T1DM clinical and research center. All study personnel will attend an investigator's meeting, which will be organized and conducted before trial initiation. All subjects will sign an informed consent form approved by Western Institutional Review Board (WIRB) and the Colorado Multiple Institutional Review Board (COMIRB).

Study results will be presented at national and international conferences including the Scientific Sessions of the American Diabetes Association (ADA), EASD, ATTD, etc. The Principal Investigator may submit study results for publishing to peer reviewed scientific journals, particularly within the fields of diabetes and endocrinology.

**Table 1:** Study Procedures for Subjects

| Visit | 1 | 2 | 3 - 13 | 14 |
|---|---|---|---|---|
| **Study Week** | **0** | **1** | **5-40** | **44-52** |
| Informed Consent and Eligibility Criteria | X | | | |
| Demographic Data, Medical History | X | | | |
| Concomitant Medications | X | X | X | X |
| Adverse Events | X | X | X | X |
| Physical Exam | X | X | X* | X |
| Urine Pregnancy Test | | X | | |
| A1C | X | X | X | X |
| Vital Signs, Height, Weight | X | X | X | X |
| Record Basal, Bolus, Total Insulin Dose** | X | X | X | X |
| SMBG Questionnaire | | X | X* | X |
| Glucose Meter Download | X | X | X | X |
| Insulin Pump Download (if applicable) | X | X | X | X |
| **Dexcom™ CGM Training and Education (if needed, Groups 1 & 2)** | | X | | |
| CGM sensor insertion | | X | X | |
| CGM Removal/ Download | | X | X | X |
| Record interventions of a 3rd party for a subject's high or low glucose levels | | X | X | X |
| Hypoglycemic Fear Questionnaire | | X | X* | X |
| BDC Pregnancy CGM Post-Partum Questionnaire | | | | X |

* Once per trimester.

** From insulin pump download or record of patient's MDI insulin use.

**Table 2:** Study Procedures for Followers

| Visit | 1 | 2 | 3 - 13 | 14 |
|---|---|---|---|---|
| **Study Week** | **0** | **1** | **5-40** | **44-52** |
| Informed Consent and Eligibility Criteria | X | | | |
| Follower Questionnaire | | | X* | X |

* Once per month.

**Figure 1 Study design:**

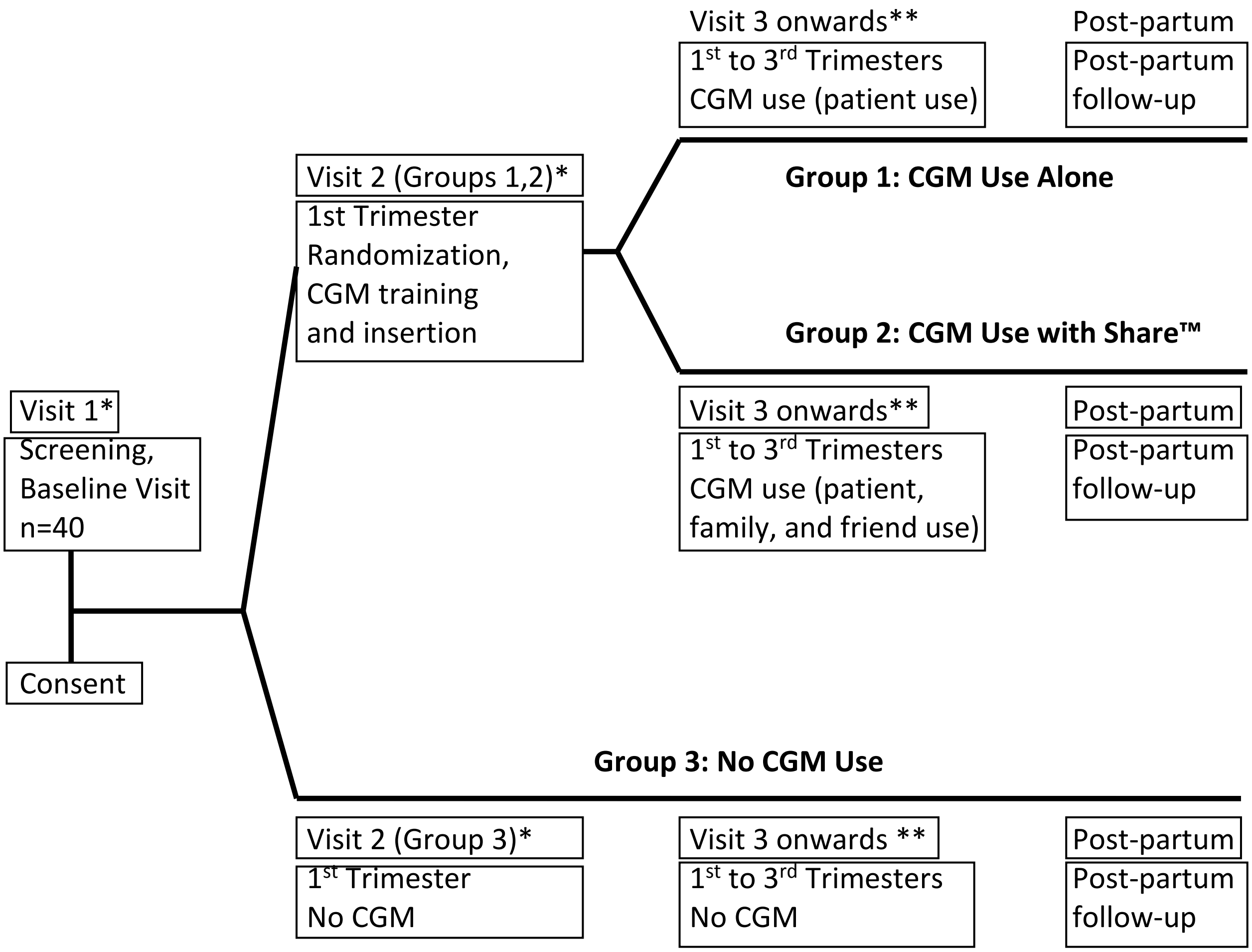


*Visit 1 will apply for women screened at a pre-conception counseling visit. Women screened in the 1st trimester may combine visits 1 and 2.

**Visits 3 through 13 for pregnancy care and study follow-up.

## Appendix A

| The **Dexcom G4 Platinum® CGM system** should be used: | About the Share™ technology |
|---|---|
| ★ For measuring interstitial glucose levels from the sensor tip which is inserted into the skin of the abdomen or forearm.<br>★ With calibration with measurements of blood sugar levels from fresh capillary whole blood samples taken from the fingertip, palm (at the base of the thumb), or forearm.<br><br>For use at home by persons with diabetes, or in a clinical setting by healthcare professionals, as an aid to monitor the effectiveness of diabetes control. | ★ **Remote monitoring capacity:** Bluetooth® Low Energy technology.<br>★ **Compatibility with:** iPhone versions 4S through 6 Plus, iPod touch 5th Gen, iPad versions 3 and 4, iPad Air and Air 2, iPad Mini versions 1 through 3.<br>★ **Apps:** Dexcom Share and Dexcom Follow both require internet connectivity<br>★ **Number of followers allowed:** 5.<br>★ **Information sharing:** Applies when the app is running in the foreground or background, as long as the app is open.<br>★ **Access to trend graph and notification settings:** Applies when the Sharer sets access to the follower, Sharer can change access to the trend graph, Follower can modify the notification settings. |

# Appendix B

## Baseline Participant Questionnaire

Subject ID: ________________________________ Study Visit: (dd/mmm/yyyy) : _____/_____/_____

**DEMOGRAPHIC INFORMATION**

Date of Birth ______________
(dd/mmm/yyyy)

Ethnicity
- ☐ Asian/Oriental
- ☐ African American
- ☐ Caucasian
- ☐ Hispanic or Latino
- ☐ Other If other, specify ______________

Education
- ☐ Grade school
- ☐ High School/GED
- ☐ Some College
- ☐ Associates degree
- ☐ Bachelor's Degree
- ☐ Master's degree
- ☐ Other If other, specify ______________

Marital Status
- ☐ Single
- ☐ Married
- ☐ Widowed
- ☐ Other If other, specify ______________

**DIABETES HISTORY**

Duration of Diabetes: ______________________

Immediate family history of diabetes? Yes ☐ No ☐

Method of Insulin delivery:
- ☐ MDI
- ☐ Pump Therapy
- ☐ VGO Insulin Therapy

Type and Average Dose of Insulin Used:
Basal: _______________________ Dose______
Bolus:_______________________ Dose______

Other Diabetes medications? __________
If yes, specify ________________________________________________________

CGM Use: ☐ Current CGM user If yes, duration of previous use ____________
☐ Past CGM user
☐ Never used CGM

## DIABETIC COMPLICATIONS

Diabetic Retinopathy? Yes ☐ No ☐ Unknown ☐

Diabetic sensory or motor neuropathy? Yes ☐ No ☐ Unknown ☐

Diabetic autonomic neuropathy? Yes ☐ No ☐ Unknown ☐

Diabetic nephropathy? (including micro-albuminuria) Yes ☐ No ☐ Unknown ☐

If yes, please specify the most recent event diagnosed at the time of patient inclusion:

Impaired renal function (estimated GFR by MDRD below 60mL/min) ☐

Micro-albuminuria (30-299mcg per mg creatinine) ☐

Overt proteinuria (equal to or above 300 mcg per mg creatinine) ☐

High Cholesterol? Yes ☐ No ☐ Unknown ☐

High Blood Pressure? Yes ☐ No ☐ Unknown ☐

## PRE-EXISTING CONDITIONS

Does the subject have any pre-existing conditions? Yes ☐ No ☐

If yes, list below

______________________________________________________________________________
______________________________________________________________________________
______________________________________________________________________________
______________________________________________________________________________

## CONCOMITANT THERAPY

Is the subject taking any medications other than insulin? Yes ☐ No ☐ If yes, list below

______________________________________________________________________________
______________________________________________________________________________
______________________________________________________________________________
______________________________________________________________________________
______________________________________________________________________________

## PREVIOUS OBSTETRIC HISTORY

Past pregnancy? Yes ☐ No ☐

If no, skip to next section.

Fill out information for each pregnancy. If there are more than 4 pregnancies, continue on another sheet.

**First Pregnancy**

Outcome Date ____________
(dd/mmm/yyyy)

Delivery type:

Vaginal ☐ If yes: Spontaneous ☐ Induced ☐ Breech ☐ Vacuum ☐ Forceps
VBAC ☐ If yes: Spontaneous ☐ Induced ☐
Cesarean Section ☐ If yes: Low Transverse ☐ Low Vertical ☐ Classical ☐ Unspecified ☐
Abortion ☐ If yes: Spontaneous ☐ Therapeutic ☐
Tubal/ectopic ☐

Gestational Age ____________ Birth Weight ____________

Preterm labor during pregnancy? Yes ☐ No ☐

Child is still living? Yes ☐ No ☐

Pregnancy complications? Yes ☐ No ☐

If yes, check all that apply:

Pregnancy-induced hypertension ☐
Eclampsia/Pre-eclampsia ☐
Large for gestational age baby ☐
Small for gestational age baby ☐
Hypoglycemic seizure ☐
Diabetic Ketoacidosis ☐
Progression of diabetic complication(s) ☐
Other ☐ Specify ______________________

Labor complications? Yes ☐ No ☐

If yes, check all that apply:

Fetal Intolerance ☐
Eclampsia/Pre-eclampsia ☐
Seizures during Labor ☐
Aburptio Placenta ☐
Shoulder Dystocia ☐
Failure to Progress in First State ☐
Neonatal Hypoglycemia ☐
Anesthetic Complications ☐
Dysfunctional Labor ☐
Cephalopelvic Disproportion ☐
Placenta Previa ☐
Excessive Bleeding ☐
Failure to Progress in Second State ☐
Other ☐ Specify __________________________

**Second Pregnancy**

Outcome Date ____________
(dd/mmm/yyyy)

Delivery type:

Vaginal ☐ If yes: Spontaneous ☐ Induced ☐ Breech ☐ Vacuum ☐ Forceps
VBAC ☐ If yes: Spontaneous ☐ Induced ☐
Cesarean Section ☐ If yes: Low Transverse ☐ Low Vertical ☐ Classical ☐ Unspecified ☐
Abortion ☐ If yes: Spontaneous ☐ Therapeutic ☐
Tubal/ectopic ☐

Gestational Age ____________ Birth Weight ____________

Preterm labor during pregnancy? Yes ☐ No ☐

Child is still living? Yes ☐ No ☐

Pregnancy complications? Yes ☐ No ☐

If yes, check all that apply:

- Pregnancy-induced hypertension ☐
- Eclampsia/Pre-eclampsia ☐
- Large for gestational age baby ☐
- Small for gestational age baby ☐
- Hypoglycemic seizure ☐
- Diabetic Ketoacidosis ☐
- Progression of diabetic complication(s) ☐
- Other ☐ Specify ______________________

Labor complications? Yes ☐ No ☐

If yes, check all that apply:

- Fetal Intolerance ☐
- Eclampsia/Pre-eclampsia ☐
- Seizures during Labor ☐
- Aburptio Placenta ☐
- Shoulder Dystocia ☐
- Failure to Progress in First State ☐
- Neonatal Hypoglycemia ☐
- Anesthetic Complications ☐
- Dysfunctional Labor ☐
- Cephalopelvic Disproportion ☐
- Placenta Previa ☐
- Excessive Bleeding ☐
- Failure to Progress in Second State ☐
- Other ☐ Specify __________________________

**Third Pregnancy**

Outcome Date ____________
(dd/mmm/yyyy)

Delivery type:

- Vaginal ☐ If yes: Spontaneous ☐ Induced ☐ Breech ☐ Vacuum ☐ Forceps
- VBAC ☐ If yes: Spontaneous ☐ Induced ☐
- Cesarean Section ☐ If yes: Low Transverse ☐ Low Vertical ☐ Classical ☐ Unspecified ☐
- Abortion ☐ If yes: Spontaneous ☐ Therapeutic ☐
- Tubal/ectopic ☐

Gestational Age ____________ Birth Weight ____________

Preterm labor during pregnancy? Yes ☐ No ☐

Child is still living? Yes ☐ No ☐

Pregnancy complications? Yes ☐ No ☐

If yes, check all that apply:

- Pregnancy-induced hypertension ☐
- Eclampsia/Pre-eclampsia ☐
- Large for gestational age baby ☐
- Small for gestational age baby ☐
- Hypoglycemic seizure ☐
- Diabetic Ketoacidosis ☐
- Progression of diabetic complication(s) ☐
- Other ☐ Specify ______________________

Labor complications? Yes ☐ No ☐

If yes, check all that apply:

- Fetal Intolerance ☐
- Eclampsia/Pre-eclampsia ☐
- Seizures during Labor ☐
- Aburptio Placenta ☐
- Shoulder Dystocia ☐
- Failure to Progress in First State ☐
- Neonatal Hypoglycemia ☐
- Anesthetic Complications ☐
- Dysfunctional Labor ☐
- Cephalopelvic Disproportion ☐
- Placenta Previa ☐
- Excessive Bleeding ☐
- Failure to Progress in Second State ☐
- Other ☐ Specify __________________________

**Fourth Pregnancy**

Outcome Date ____________
(dd/mmm/yyyy)

Delivery type:

Vaginal ☐ If yes: Spontaneous ☐ Induced ☐ Breech ☐ Vacuum ☐ Forceps

VBAC ☐ If yes: Spontaneous ☐ Induced ☐

Cesarean Section ☐ If yes: Low Transverse ☐ Low Vertical ☐ Classical ☐ Unspecified ☐

Abortion ☐ If yes: Spontaneous ☐ Therapeutic ☐

Tubal/ectopic ☐

Gestational Age ____________ Birth Weight ____________

Preterm labor during pregnancy? Yes ☐ No ☐

Child is still living? Yes ☐ No ☐

Pregnancy complications? Yes ☐ No ☐

If yes, check all that apply:

Pregnancy-induced hypertension ☐
Eclampsia/Pre-eclampsia ☐
Large for gestational age baby ☐
Small for gestational age baby ☐
Hypoglycemic seizure ☐
Diabetic Ketoacidosis ☐
Progression of diabetic complication(s) ☐
Other ☐ Specify ______________________

Labor complications? Yes ☐ No ☐

If yes, check all that apply:

Fetal Intolerance ☐
Eclampsia/Pre-eclampsia ☐
Seizures during Labor ☐
Aburptio Placenta ☐
Shoulder Dystocia ☐
Failure to Progress in First State ☐
Neonatal Hypoglycemia ☐
Anesthetic Complications ☐
Dysfunctional Labor ☐
Cephalopelvic Disproportion ☐
Placenta Previa ☐
Excessive Bleeding ☐
Failure to Progress in Second State ☐
Other ☐ Specify __________________________

## ALCOHOL AND TOBACCO CONSUMPTION HABITS

Past cigarette use? Yes ☐ No ☐

Current cigarette use? Yes ☐ No ☐

If yes, usage duration ____________

Alcohol use:
Never ☐
Occasionally (less than monthly) ☐
At least monthly ☐
At least weekly ☐
At least daily ☐

How many standard drinks* containing alcohol does subject have on a typical day when drinking?

1 or 2 ☐ Greater than 2 ☐

*standard drink: 1 pint/bottle of beer, 1 glass of wine, 1 shot of hard liquor

**Appendix C**

# Hypoglycemic Fear Survey

Subject ID: ______________________ Clinic Visit: (dd/mmm/yyyy) : _____/_____/_____

Visit (check one): __Baseline __First Trimester __Second Trimester __Third Trimester __Post-Partum

This survey is intended to find out more about how low blood sugar makes people feel Please answer the following questions as frankly as possible.

**I. Behavior.** Below is a list of things people with diabetes sometimes do in order to avoid low blood sugar. Read each item carefully. Circle one of the numbers to the right that best describes what you do during your daily routine to avoid low blood sugar.

| | Never | Rarely | Sometimes | Often | Very often |
|---|---|---|---|---|---|
| 1. Eat large snacks at bedtime | 1 | 2 | 3 | 4 | 5 |
| 2. Avoid being alone when my sugar is likely to be low | 1 | 2 | 3 | 4 | 5 |
| 3. If test urine, spill a little sugar to be on the safe side. If test blood glucose, run a little high to be on the safe side | 1 | 2 | 3 | 4 | 5 |
| 4. Keep my sugar higher when I will be alone for a while | 1 | 2 | 3 | 4 | 5 |
| 5. Eat something as soon as I feel the first sign of low blood sugar | 1 | 2 | 3 | 4 | 5 |
| 6. Reduce my medication (insulin/pills) when I think my sugar is too low | 1 | 2 | 3 | 4 | 5 |
| 7. Keep my blood sugar higher when I plan to be in a long meeting or at a party | 1 | 2 | 3 | 4 | 5 |
| 8. Carry fast-acting sugar with me | 1 | 2 | 3 | 4 | 5 |
| 9. Avoid a lot of exercise when I think my sugar is low | 1 | 2 | 3 | 4 | 5 |
| 10. Check my sugar often when I plan to be in a long meeting or go out to a party | 1 | 2 | 3 | 4 | 5 |

**II. Worry.** Below is a list of concerns people with diabetes sometimes have. Please read each item carefully (do not skip any). Circle one of the numbers to the right that best describes how often you worry about each item because of low blood sugar.

| | Never | Rarely | Sometimes | Often | Very often |
|---|---|---|---|---|---|
| 11. Not recognizing/realizing I am having a reaction | 1 | 2 | 3 | 4 | 5 |
| 12. Not having food, fruit, or juice with me | 1 | 2 | 3 | 4 | 5 |
| 13. Feeling dizzy or passing out in public | 1 | 2 | 3 | 4 | 5 |
| 14. Having a reaction while asleep | 1 | 2 | 3 | 4 | 5 |
| 15. Embarrassing myself or my friends/family in a social situation | 1 | 2 | 3 | 4 | 5 |
| 16. Having a reaction while alone | 1 | 2 | 3 | 4 | 5 |
| 17. Appearing stupid or drunk | 1 | 2 | 3 | 4 | 5 |
| 18. Losing control | 1 | 2 | 3 | 4 | 5 |
| 19. No one being around to help me during a reaction | 1 | 2 | 3 | 4 | 5 |
| 20. Having a reaction while driving | 1 | 2 | 3 | 4 | 5 |
| 21. Making a mistake or having an accident at work | 1 | 2 | 3 | 4 | 5 |
| 22. Getting a bad evaluation at work because of something that happens when my sugar is low | 1 | 2 | 3 | 4 | 5 |
| 23. Having seizures or convulsions | 1 | 2 | 3 | 4 | 5 |
| 24. Difficulty thinking clearly when responsible for others (children, elderly, etc.) | 1 | 2 | 3 | 4 | 5 |
| 25. Developing long-term complications from frequent low blood sugar | 1 | 2 | 3 | 4 | 5 |
| 26. Feeling lightheaded or faint | 1 | 2 | 3 | 4 | 5 |
| 27. Having an insulin reaction | 1 | 2 | 3 | 4 | 5 |

### Appendix D

# SMBG (Self-Monitoring Blood Glucose) Questionnaire

Subject ID: ______________________ Clinic Visit: (dd/mmm/yyyy) : _____/_____/_____

Gestational Age: __8 to 12 weeks __20 to 24 weeks __32 to 36 weeks __Post-Partum

| DAY | DATE | TIME POINT | BLOOD GLUCOSE BEFORE MEAL (OR BEDTIME) | SHORT-ACTING INSULIN DOSE (UNITS) | BLOOD GLUCOSE 2 HOURS AFTER MEAL |
|---|---|---|---|---|---|
| 1 | | Breakfast | | | |
| | | Lunch | | | |
| | | Dinner | | | |
| | | Bedtime | | | |
| 2 | | Breakfast | | | |
| | | Lunch | | | |
| | | Dinner | | | |
| | | Bedtime | | | |
| 3 | | Breakfast | | | |
| | | Lunch | | | |
| | | Dinner | | | |
| | | Bedtime | | | |
| 4 | | Breakfast | | | |
| | | Lunch | | | |
| | | Dinner | | | |
| | | Bedtime | | | |
| 5 | | Breakfast | | | |
| | | Lunch | | | |
| | | Dinner | | | |
| | | Bedtime | | | |
| 6 | | Breakfast | | | |
| | | Lunch | | | |
| | | Dinner | | | |
| | | Bedtime | | | |
| 7 | | Breakfast | | | |
| | | Lunch | | | |
| | | Dinner | | | |
| | | Bedtime | | | |

**Appendix E**

# Follower Questionnaire

Subject ID: ______________________ Clinic Visit: (dd/mmm/yyyy) : _____/_____/_____

Gestational Age of Pregnant Participant:

___4 to 8 weeks ___8 to 12 weeks ___12 to 16 weeks ___16 to 20 weeks ___20 to 24 weeks

___24 to 28 weeks ___28 to 32 weeks ___32 to 36 weeks ___36 to 40 weeks ___40 to 42 weeks

__4 to 12 weeks post-partum

## LOW GLUCOSE QUESTIONS

How many times were you alerted to low glucose levels this month?

☐ Never ☐ 1-5x mo ☐ 1-5 x weekly ☐ Daily ☐ More than 1 x daily ☐ N/A

Did you intervene to help your pregnant partner with low glucose levels this month?

☐ Yes ☐ No

If yes, how often?

☐ Never ☐ 1-5x mo ☐ 1-5 x weekly ☐ Daily ☐ More than 1 x daily ☐ N/A

If yes, what was the nature of the intervention? Check all that apply.

☐ Gave glucose tablets/gel ☐ Gave glucagon injection

☐ Gave food or drink with carbohydrates ☐ Called health care provider

☐ Checked blood glucose level ☐ Called emergency services (like 911)

☐ Other (Please describe: ________________________________________________)

How many times did your pregnant partner's low glucose levels interfere with her daily activities this month?

☐ Never ☐ 1-5x mo ☐ 1-5 x weekly ☐ Daily ☐ More than 1 x daily ☐ N/A

## HIGH GLUCOSE QUESTIONS

How many times were you alerted to high glucose levels this month?

☐ Never ☐ 1-5x mo ☐ 1-5 x weekly ☐ Daily ☐ More than 1 x daily ☐ N/A

Did you intervene to help your pregnant partner with high glucose levels this month?

☐ Yes ☐ No

If yes, how often?

☐ Never ☐ 1-5x mo ☐ 1-5 x weekly ☐ Daily ☐ More than 1 x daily ☐ N/A

If yes, what was the nature of the intervention? Check all that apply.

☐ Gave insulin shot

☐ Gave insulin through an insulin pump

☐ Checked blood glucose level

☐ Checked ketone level

☐ Called health care provider

☐ Called emergency services (like 911)

☐ Other (Please describe: _______________________________________________)

How many times did your pregnant partner's high glucose levels interfere with her daily activities this month?

☐ Never ☐ 1-5x mo ☐ 1-5 x weekly ☐ Daily ☐ More than 1 x daily ☐ N/A

Appendix F

# BDC Pregnancy CGM Post-Partum Questionnaire

Subject ID: ____________________ Clinic Visit: (dd/mmm/yyyy) : _____/_____/_____

Post-Partum Week: ___4 to 6 weeks ___6 to 8 weeks ___8 to 10 weeks ___10 to 12 weeks

---

Pregnancy Outcome Date __________
(dd/mmm/yyyy)

Gestational Age __________ Birth Weight __________ Birth Length __________

Child is still living? ☐ Yes ☐ No Baby's Sex ☐ Male ☐ Female

Preterm labor during pregnancy? ☐ Yes ☐ No
If yes, please describe, including dates of hospital admissions: ______________________
________________________________________________________________________

**Delivery type:**

Vaginal ☐ If yes: Spontaneous ☐ Induced ☐ Breech ☐ Vacuum ☐ Forceps
VBAC ☐ If yes: Spontaneous ☐ Induced ☐
Cesarean Section ☐ If yes: Low Transverse ☐ Low Vertical ☐ Classical ☐ Unspecified ☐

**Pregnancy complications?** ☐ Yes ☐ No
If yes, check all that apply:

☐ Placenta previa ☐ Placenta accreta ☐ Incompetent cervix
☐ Hyperemesis gravidarum ☐ Proteinuria ☐ Hypoglycemic seizure
☐ Pregnancy-induced hypertension ☐ Oligohydramnios ☐ Polyhydramnios
☐ Pre-eclampsia ☐ Eclampsia ☐ Diabetic ketoacidosis
☐ HELLP syndrome ☐ Progression of diabetic complications
☐ Small for gestational age baby ☐ Large for gestational age baby
☐ Other ____________________________________________________________

**Labor complications?** ☐ Yes ☐ No
If yes, check all that apply:

☐ Fetal intolerance ☐ Anesthetic complications ☐ Pre-eclampsia
☐ Eclampsia ☐ Dysfunctional labor ☐ Seizures during labor
☐ Cephalopelvic disproportion ☐ Placental abruption ☐ Placenta previa
☐ Shoulder dystocia ☐ Breech delivery ☐ Nuchal cord
☐ Excessive bleeding ☐ Failure to progress in first state
☐ Failure to progress in second state ☐ Other ________________________________

**Neonatal complications?** ☐ Yes ☐ No
If yes, check all that apply:

☐ Birth trauma/injury ☐ Hypoglycemia ☐ Asphyxia
☐ Jaundice ☐ Congenital anomalies ________________________

☐ Other ___________________________________________________________

Was the baby admitted to the Neonatal Intensive Care Unit (NICU) for any length of time during your hospital stay? ☐ Yes ☐ No

If yes, please describe: ____________________________________________________
_____________________________________________________________________
_____________________________________________________________________
_____________________________________________________________________

**Are you or did you ever breastfeed after your baby was delivered?** ☐ Yes ☐ No

If yes, check all that apply:

- ☐ Breastfed within the <u>first week</u> of baby's birth and not afterwards
- ☐ Breastfed within the <u>first month</u> of baby's birth and not afterwards
- ☐ Breastfed <u>for the first 2 months</u> after baby's birth and not afterwards
- ☐ Breastfed <u>for the first 3 months</u> after baby's birth and not afterwards
- ☐ Breastfed <u>for the first 3 months</u> after baby's birth and <u>still breastfeeding</u>
- ☐ Breastfed exclusively
- ☐ Breastfed and formula-fed the baby

**Questions about glucose variability:**

Have you had any glucose values below 50 within the last month? ☐ Yes ☐ No

If yes, how often on average?

- ☐ Every day
- ☐ A few times per week
- ☐ A few times per month
- ☐ Once

Over the last month did you have to do something to correct a low glucose (<50 mg/dL) or did someone else have to intervene for a low glucose on your behalf? ☐ Yes ☐ No

If yes, check all that apply:

- ☐ Consume glucose tablets or glucose gel
- ☐ Eat or drink something with carbohydrates
- ☐ Call health care provider for help
- ☐ Receive an injection of glucagon
- ☐ Receive an infusion of dextrose (glucose through a vein)
- ☐ Call to emergency services (like 911)
- ☐ Stay in the hospital for observation and/or treatment

If yes, when? _____________ For how long? ______________

Have you had any glucose values above 300 within the last month? ☐ Yes ☐ No

If yes, how often on average?

- ☐ Every day
- ☐ A few times per week
- ☐ A few times per month
- ☐ Once

Over the last month did you have to do something unusual (besides taking extra insulin) for a very high glucose (<300 mg/dL) or did someone else have to intervene for a very high glucose on your behalf? ☐ Yes ☐ No

If yes, check all that apply:

☐ Take multiple insulin boluses within 6 hours to bring the glucose down
☐ Change an infusion set earlier than anticipated
☐ Drink extra amounts of fluids
☐ Call health care provider for help
☐ Call to emergency services (like 911)
☐ Receive insulin and/or fluids through a vein
☐ Stay in the hospital for observation and/or treatment

If yes, when? ______________ For how long? _______________

## References:

1. Center for Disease Control and Prevention. National Diabetes Statistics Report: Estimates of Diabetes and Its Burden in the United States, 2014. Atlanta, GA: U.S. Department of Health and Human Services, 2014.
2. Kitzmiller JL, Block JM, Brown FM, Catalano PM, Conway DL, Coustan DR, et al. Managing preexisting diabetes for pregnancy: summary of evidence and consensus recommendations for care. Diabetes care. 2008;31(5):1060-79.
3. Kerssen A, de Valk HW, Visser GH. The Continuous Glucose Monitoring System during pregnancy of women with type 1 diabetes mellitus: accuracy assessment. Diabetes technology & therapeutics. 2004;6(5):645-51.
4. Secher AL, Stage E, Ringholm L, Barfred C, Damm P, Mathiesen ER. Real-time continuous glucose monitoring as a tool to prevent severe hypoglycaemia in selected pregnant women with Type 1 diabetes - an observational study. Diabetic medicine : a journal of the British Diabetic Association. 2014;31(3):352-6.
5. Murphy HR, Rayman G, Lewis K, Kelly S, Johal B, Duffield K, et al. Effectiveness of continuous glucose monitoring in pregnant women with diabetes: randomised clinical trial. Bmj. 2008;337:a1680.
6. Murphy HR. Integrating educational and technological interventions to improve pregnancy outcomes in women with diabetes. Diabetes, obesity & metabolism. 2010;12(2):97-104.
7. Petrovski G, Dimitrovski C, Bogoev M, Milenkovic T, Ahmeti I, Bitovska I. Is there a difference in pregnancy and glycemic outcome in patients with type 1 diabetes on insulin pump with constant or intermittent glucose monitoring? A pilot study. Diabetes technology & therapeutics. 2011;13(11):1109-13.
