# Supplementary material for: Continuous glucose monitor use with and without remote monitoring in pregnant women with type 1 diabetes: A pilot study
Source: PLoS One. 2020 Apr 16;15(4):e0230476. doi: 10.1371/journal.pone.0230476 (PMC7162510; doi:10.1371/journal.pone.0230476)
Supplement: S1 File — (PDF) [file pone.0230476.s003.pdf]

Pilot CGM Project in Pregnancy

Baseline Participant Questionnaire

Subject ID: ________________________________ Study Visit: (dd/mmm/yyyy) : _____/_____/_____

DEMOGRAPHIC INFORMATION

Date of Birth ______________
(dd/mmm/yyyy)

Ethnicity
- ☐ Asian/Oriental
- ☐ African American
- ☐ Caucasian
- ☐ Hispanic or Latino
- ☐ Other If other, specify ______________

Education
- ☐ Grade school
- ☐ High School/GED
- ☐ Some College
- ☐ Associates degree
- ☐ Bachelor's Degree
- ☐ Master's degree
- ☐ Other If other, specify ______________

Marital Status
- ☐ Single
- ☐ Married
- ☐ Widowed
- ☐ Other If other, specify ______________

DIABETES HISTORY

Duration of Diabetes: ______________________

Immediate family history of diabetes? Yes ☐ No ☐

Method of Insulin delivery:
- ☐ MDI
- ☐ Pump Therapy
- ☐ VGO Insulin Therapy

Type and Average Dose of Insulin Used:
Basal: ______________________ Dose______
Bolus:______________________ Dose______

Other Diabetes medications? __________
If yes, specify ____

CGM Use: ☐ Current CGM user If yes, duration of previous use ____________
☐ Past CGM user
☐ Never used CGM

DIABETIC COMPLICATIONS

Diabetic Retinopathy? Yes ☐ No ☐ Unknown ☐

Diabetic sensory or motor neuropathy? Yes ☐ No ☐ Unknown ☐

Diabetic autonomic neuropathy? Yes ☐ No ☐ Unknown ☐

Diabetic nephropathy? (including micro-albuminuria) Yes ☐ No ☐ Unknown ☐

If yes, please specify the most recent event diagnosed at the time of patient inclusion:

Impaired renal function (estimated GFR by MDRD below 60mL/min) ☐

Micro-albuminuria (30-299mcg per mg creatinine) ☐

Overt proteinuria (equal to or above 300 mcg per mg creatinine) ☐

High Cholesterol? Yes ☐ No ☐ Unknown ☐

High Blood Pressure? Yes ☐ No ☐ Unknown ☐

PRE-EXISTING CONDITIONS

Does the subject have any pre-existing conditions? Yes ☐ No ☐

If yes, list below

____
____
____
____

CONCOMITANT THERAPY

Is the subject taking any medications other than insulin? Yes ☐ No ☐ If yes, list below

____
____
____
____
____

PREVIOUS OBSTETRIC HISTORY

Past pregnancy? Yes ☐ No ☐

If no, skip to next section.

Fill out information for each pregnancy. If there are more than 4 pregnancies, continue on another sheet.

First Pregnancy

Outcome Date ____________
(dd/mmm/yyyy)

Delivery type:

Vaginal ☐ If yes: Spontaneous ☐ Induced ☐ Breech ☐ Vacuum ☐ Forceps
VBAC ☐ If yes: Spontaneous ☐ Induced ☐
Cesarean Section ☐ If yes: Low Transverse ☐ Low Vertical ☐ Classical ☐ Unspecified ☐
Abortion ☐ If yes: Spontaneous ☐ Therapeutic ☐
Tubal/ectopic ☐

Gestational Age ____________ Birth Weight ____________

Preterm labor during pregnancy? Yes ☐ No ☐

Child is still living? Yes ☐ No ☐

Pregnancy complications? Yes ☐ No ☐

If yes, check all that apply:

Pregnancy-induced hypertension ☐
Eclampsia/Pre-eclampsia ☐
Large for gestational age baby ☐
Small for gestational age baby ☐
Hypoglycemic seizure ☐
Diabetic Ketoacidosis ☐
Progression of diabetic complication(s) ☐
Other ☐ Specify ______________________

Labor complications? Yes ☐ No ☐

If yes, check all that apply:

Fetal Intolerance ☐
Eclampsia/Pre-eclampsia ☐
Seizures during Labor ☐
Aburptio Placenta ☐
Shoulder Dystocia ☐
Failure to Progress in First State ☐
Neonatal Hypoglycemia ☐
Anesthetic Complications ☐
Dysfunctional Labor ☐
Cephalopelvic Disproportion ☐
Placenta Previa ☐
Excessive Bleeding ☐
Failure to Progress in Second State ☐
Other ☐ Specify ___________________________

Second Pregnancy

Outcome Date ____________
(dd/mmm/yyyy)

Delivery type:

Vaginal ☐ If yes: Spontaneous ☐ Induced ☐ Breech ☐ Vacuum ☐ Forceps
VBAC ☐ If yes: Spontaneous ☐ Induced ☐
Cesarean Section ☐ If yes: Low Transverse ☐ Low Vertical ☐ Classical ☐ Unspecified ☐
Abortion ☐ If yes: Spontaneous ☐ Therapeutic ☐
Tubal/ectopic ☐

Gestational Age ____________ Birth Weight ____________

Preterm labor during pregnancy? Yes ☐ No ☐

Child is still living? Yes ☐ No ☐

Pregnancy complications? Yes ☐ No ☐

If yes, check all that apply:

Pregnancy-induced hypertension ☐
Eclampsia/Pre-eclampsia ☐
Large for gestational age baby ☐
Small for gestational age baby ☐
Hypoglycemic seizure ☐
Diabetic Ketoacidosis ☐
Progression of diabetic complication(s) ☐
Other ☐ Specify ______________________

Labor complications? Yes ☐ No ☐

If yes, check all that apply:

Fetal Intolerance ☐
Eclampsia/Pre-eclampsia ☐
Seizures during Labor ☐
Aburptio Placenta ☐
Shoulder Dystocia ☐
Failure to Progress in First State ☐
Neonatal Hypoglycemia ☐
Anesthetic Complications ☐
Dysfunctional Labor ☐
Cephalopelvic Disproportion ☐
Placenta Previa ☐
Excessive Bleeding ☐
Failure to Progress in Second State ☐
Other ☐ Specify __________________________

Third Pregnancy

Outcome Date ____________
(dd/mmm/yyyy)

Delivery type:

Vaginal ☐ If yes: Spontaneous ☐ Induced ☐ Breech ☐ Vacuum ☐ Forceps
VBAC ☐ If yes: Spontaneous ☐ Induced ☐
Cesarean Section ☐ If yes: Low Transverse ☐ Low Vertical ☐ Classical ☐ Unspecified ☐
Abortion ☐ If yes: Spontaneous ☐ Therapeutic ☐
Tubal/ectopic ☐

Gestational Age ____________ Birth Weight ____________

Preterm labor during pregnancy? Yes ☐ No ☐

Child is still living? Yes ☐ No ☐

Pregnancy complications? Yes ☐ No ☐

If yes, check all that apply:

Pregnancy-induced hypertension ☐
Eclampsia/Pre-eclampsia ☐
Large for gestational age baby ☐
Small for gestational age baby ☐
Hypoglycemic seizure ☐
Diabetic Ketoacidosis ☐
Progression of diabetic complication(s) ☐
Other ☐ Specify ______________________

Labor complications? Yes ☐ No ☐

If yes, check all that apply:

Fetal Intolerance ☐
Eclampsia/Pre-eclampsia ☐
Seizures during Labor ☐
Aburptio Placenta ☐
Shoulder Dystocia ☐
Failure to Progress in First State ☐
Neonatal Hypoglycemia ☐
Anesthetic Complications ☐
Dysfunctional Labor ☐
Cephalopelvic Disproportion ☐
Placenta Previa ☐
Excessive Bleeding ☐
Failure to Progress in Second State ☐
Other ☐ Specify __________________________

Fourth Pregnancy

Outcome Date ____________
(dd/mmm/yyyy)

Delivery type:

Vaginal ☐ If yes: Spontaneous ☐ Induced ☐ Breech ☐ Vacuum ☐ Forceps
VBAC ☐ If yes: Spontaneous ☐ Induced ☐
Cesarean Section ☐ If yes: Low Transverse ☐ Low Vertical ☐ Classical ☐ Unspecified ☐
Abortion ☐ If yes: Spontaneous ☐ Therapeutic ☐
Tubal/ectopic ☐

Gestational Age ____________ Birth Weight ____________

Preterm labor during pregnancy? Yes ☐ No ☐

Child is still living? Yes ☐ No ☐

Pregnancy complications? Yes ☐ No ☐
If yes, check all that apply:
Pregnancy-induced hypertension ☐
Eclampsia/Pre-eclampsia ☐
Large for gestational age baby ☐
Small for gestational age baby ☐
Hypoglycemic seizure ☐
Diabetic Ketoacidosis ☐
Progression of diabetic complication(s) ☐
Other ☐ Specify ______________________

Labor complications? Yes ☐ No ☐
If yes, check all that apply:
Fetal Intolerance ☐
Eclampsia/Pre-eclampsia ☐
Seizures during Labor ☐
Aburptio Placenta ☐
Shoulder Dystocia ☐
Failure to Progress in First State ☐
Neonatal Hypoglycemia ☐
Anesthetic Complications ☐
Dysfunctional Labor ☐
Cephalopelvic Disproportion ☐
Placenta Previa ☐
Excessive Bleeding ☐
Failure to Progress in Second State ☐
Other ☐ Specify __________________________

ALCOHOL AND TOBACCO CONSUMPTION HABITS

Past cigarette use? Yes ☐ No ☐

Current cigarette use? Yes ☐ No ☐

If yes, usage duration ____________

Alcohol use:
Never ☐
Occasionally (less than monthly) ☐
At least monthly ☐
At least weekly ☐
At least daily ☐

How many standard drinks* containing alcohol does subject have on a typical day when drinking?
1 or 2 ☐ Greater than 2 ☐

*standard drink: 1 pint/bottle of beer, 1 glass of wine, 1 shot of hard liquor

Follower Questionnaire

Subject ID: ______________________ Clinic Visit: (dd/mmm/yyyy) : _____/_____/_____

Gestational Age of Pregnant Participant:

___4 to 8 weeks ___8 to 12 weeks ___12 to 16 weeks ___16 to 20 weeks ___20 to 24 weeks

___24 to 28 weeks ___28 to 32 weeks ___32 to 36 weeks ___36 to 40 weeks ___40 to 42 weeks

__4 to 12 weeks post-partum

LOW GLUCOSE QUESTIONS

How many times were you alerted to low glucose levels this month?

☐ Never ☐ 1-5x mo ☐ 1-5 x weekly ☐ Daily ☐ More than 1 x daily ☐ N/A

Did you intervene to help your pregnant partner with low glucose levels this month?

☐ Yes ☐ No

If yes, how often?

☐ Never ☐ 1-5x mo ☐ 1-5 x weekly ☐ Daily ☐ More than 1 x daily ☐ N/A

If yes, what was the nature of the intervention? Check all that apply.

☐ Gave glucose tablets/gel ☐ Gave glucagon injection

☐ Gave food or drink with carbohydrates ☐ Called health care provider

☐ Checked blood glucose level ☐ Called emergency services (like 911)

☐ Other (Please describe: ____)

How many times did your pregnant partner's low glucose levels interfere with her daily activities this month?

☐ Never ☐ 1-5x mo ☐ 1-5 x weekly ☐ Daily ☐ More than 1 x daily ☐ N/A

HIGH GLUCOSE QUESTIONS

How many times were you alerted to high glucose levels this month?

☐ Never ☐ 1-5x mo ☐ 1-5 x weekly ☐ Daily ☐ More than 1 x daily ☐ N/A

Did you intervene to help your pregnant partner with high glucose levels this month?

☐ Yes ☐ No

If yes, how often?

☐ Never ☐ 1-5x mo ☐ 1-5 x weekly ☐ Daily ☐ More than 1 x daily ☐ N/A

If yes, what was the nature of the intervention? Check all that apply.

☐ Gave insulin shot
☐ Checked ketone level
☐ Gave insulin through an insulin pump
☐ Called health care provider
☐ Checked blood glucose level
☐ Called emergency services (like 911)
☐ Other (Please describe: ____)

How many times did your pregnant partner's high glucose levels interfere with her daily activities this month?

☐ Never ☐ 1-5x mo ☐ 1-5 x weekly ☐ Daily ☐ More than 1 x daily ☐ N/A

Pilot CGM Project in Pregnancy

BDC Pregnancy CGM Post-Partum Questionnaire

Subject ID: ______________________ Clinic Visit: (dd/mmm/yyyy) : _____/_____/_____

Post-Partum Week: __4 to 6 weeks __6 to 8 weeks __8 to 10 weeks __10 to 12 weeks

---

Pregnancy Outcome Date ___________
(dd/mmm/yyyy)

Gestational Age ___________ Birth Weight ___________ Birth Length ___________

Child is still living? ☐ Yes ☐ No Baby's Sex ☐ Male ☐ Female

Preterm labor during pregnancy? ☐ Yes ☐ No
If yes, please describe, including dates of hospital admissions: ______________________
____

Delivery type:

Vaginal ☐ If yes: Spontaneous ☐ Induced ☐ Breech ☐ Vacuum ☐ Forceps
VBAC ☐ If yes: Spontaneous ☐ Induced ☐
Cesarean Section ☐ If yes: Low Transverse ☐ Low Vertical ☐ Classical ☐ Unspecified ☐

Pregnancy complications? ☐ Yes ☐ No
If yes, check all that apply:

- ☐ Placenta previa
- ☐ Placenta accreta
- ☐ Incompetent cervix
- ☐ Hyperemesis gravidarum
- ☐ Proteinuria
- ☐ Hypoglycemic seizure
- ☐ Pregnancy-induced hypertension
- ☐ Oligohydramnios
- ☐ Polyhydramnios
- ☐ Pre-eclampsia
- ☐ Eclampsia
- ☐ Diabetic ketoacidosis
- ☐ HELLP syndrome
- ☐ Progression of diabetic complications
- ☐ Small for gestational age baby
- ☐ Large for gestational age baby
- ☐ Other ____

Labor complications? ☐ Yes ☐ No
If yes, check all that apply:

- ☐ Fetal intolerance
- ☐ Anesthetic complications
- ☐ Pre-eclampsia
- ☐ Eclampsia
- ☐ Dysfunctional labor
- ☐ Seizures during labor
- ☐ Cephalopelvic disproportion
- ☐ Placental abruption
- ☐ Placenta previa
- ☐ Shoulder dystocia
- ☐ Breech delivery
- ☐ Nuchal cord
- ☐ Excessive bleeding
- ☐ Failure to progress in first state
- ☐ Failure to progress in second state
- ☐ Other ___________________________________

Neonatal complications? ☐ Yes ☐ No
If yes, check all that apply:

- ☐ Birth trauma/injury
- ☐ Hypoglycemia
- ☐ Asphyxia
- ☐ Jaundice
- ☐ Congenital anomalies ___________________________
- ☐ Other ____

Was the baby admitted to the Neonatal Intensive Care Unit (NICU) for any length of time during your hospital stay? ☐ Yes ☐ No

If yes, please describe: ____

____

____

____

Are you or did you ever breastfeed after your baby was delivered? ☐ Yes ☐ No

If yes, check all that apply:

☐ Breastfed within the first week of baby's birth and not afterwards
☐ Breastfed within the first month of baby's birth and not afterwards
☐ Breastfed for the first 2 months after baby's birth and not afterwards
☐ Breastfed for the first 3 months after baby's birth and not afterwards
☐ Breastfed for the first 3 months after baby's birth and still breastfeeding
☐ Breastfed exclusively
☐ Breastfed and formula-fed the baby

Questions about glucose variability:

Have you had any glucose values below 50 within the last month? ☐ Yes ☐ No

If yes, how often on average?

☐ Every day
☐ A few times per week
☐ A few times per month
☐ Once

Over the last month did you have to do something to correct a low glucose (<50 mg/dL) or did someone else have to intervene for a low glucose on your behalf? ☐ Yes ☐ No

If yes, check all that apply:

☐ Consume glucose tablets or glucose gel
☐ Eat or drink something with carbohydrates
☐ Call health care provider for help
☐ Receive an injection of glucagon
☐ Receive an infusion of dextrose (glucose through a vein)
☐ Call to emergency services (like 911)
☐ Stay in the hospital for observation and/or treatment

If yes, when? _____________ For how long? ______________

Have you had any glucose values above 300 within the last month? ☐ Yes ☐ No

If yes, how often on average?

☐ Every day
☐ A few times per week
☐ A few times per month
☐ Once

Over the last month did you have to do something unusual (besides taking extra insulin) for a very high glucose (<300 mg/dL) or did someone else have to intervene for a very high glucose on your behalf? ☐ Yes ☐ No

If yes, check all that apply:

- ☐ Take multiple insulin boluses within 6 hours to bring the glucose down
- ☐ Change an infusion set earlier than anticipated
- ☐ Drink extra amounts of fluids
- ☐ Call health care provider for help
- ☐ Call to emergency services (like 911)
- ☐ Receive insulin and/or fluids through a vein
- ☐ Stay in the hospital for observation and/or treatment

If yes, when? _____________ For how long? ______________
